# Predictors of short- and long-term adherence with a Mediterranean-type diet intervention: the PREDIMED randomized trial

**DOI:** 10.1186/s12966-016-0394-6

**Published:** 2016-06-14

**Authors:** Mary Kathryn Downer, Alfredo Gea, Meir Stampfer, Ana Sánchez-Tainta, Dolores Corella, Jordi Salas-Salvadó, Emilio Ros, Ramón Estruch, Montserrat Fitó, Enrique Gómez-Gracia, Fernando Arós, Miquel Fiol, Francisco Jose Garcia De-la-Corte, Lluís Serra-Majem, Xavier Pinto, Josep Basora, José V. Sorlí, Ernest Vinyoles, Itziar Zazpe, Miguel-Ángel Martínez-González

**Affiliations:** Department of Epidemiology, Harvard T.H. Chan School of Public Health, 677 Huntington Avenue, Boston, MA 02115 USA; Department of Preventive Medicine and Public Health, Faculty of Medicine, University of Navarra, C/Irunlarrea, No. 1. Research Building, 2nd floor, Local 2520, 31008 Pamplona, Navarra Spain; Channing Division of Network Medicine, 181 Longwood Avenue, Room 345, Boston, MA 02115 USA; Department of Preventive Medicine, University of Valencia, AVDA,Vicente Blasco Ibanez, 15, 46010 Valencia, Spain; Human Nutrition Unit, Biochemistry and Biotechnology Department, IISPV, Hospital Universitari de Sant Joan de Reus, Universitat Rovira i Virgili, C/Sant Llorenç, 21, 43201 Reus, Spain; Lipid Clinic, Department of Endocrinology and Nutrition, Institut d’Investigacions Biomediques August Pi Sunyer (IDIBAPS), Hospital Clinic, University of Barcelona, C/Villarroel, 170, 08036 Barcelona, Spain; Department of Internal Medicine, Institut d’Investigacions Biomediques August Pi Sunyer (IDIBAPS), Hospital Clinic, University of Barcelona, C/Villarroel, 170, 08036 Barcelona, Spain; Cardiovascular and Nutrition Research Group, Institut de Recerca Hospital del Mar, Carrer Dr. Aiguader, 88, 08003 Barcelona, Spain; Department of Preventive Medicine, University of Malaga, Campus de Teatinos, 29071 Malaga, Spain; Department of Cardiology, University Hospital of Araba, C/Jose Atxotegi, s/n, 01009 Vitoria-Gasteiz, Araba Spain; Palma Institute of Health Research (IdISPa), University of Balearic Islands and Hospital Son Espases, Carretera de Valldemossa, 79, 07120 Palma, Illes Balears Spain; Department of Family Medicine, Research Unit, Distrito Sanitario Atencion Primaria Sevilla, Avda. de Jerez s/n, 41007 Sevilla, Spain; Research Institute of Biomedical and Health Sciences, University of Las Palmas de Gran Canaria, Juan De Quesada 30, 35001 Las Palmas, Spain; Head of Lipid and Vascular Risk Unit, Internal Medicine Department, Hospital Universitari de Bellvitge-IDIBELL. Universidad de Barcelona, C/Freixa Larga s/n, 08907 - Hospitalet de Llobregat, Barcelona, Spain; Human Nutrition Unit, Biochemistry and Biotechnology Department, IISPV Universitat Rovira i Virgili, C/Sant Llorenç, 21 Planta baja del edificio 4 de la Facultat de Medicina i Ciències de la Salut, 43201 Reus, Tarragona Spain; Jordi Gol Primary Care Research Institute, Gran Via de les Corts Catalanes 587, àtic, 08007 Barcelona, Spain; Centro de Investigación Biomédica en Red Fisiopatología de la Obesidad y Nutrición (CIBERobn), Instituto de Salud Carlos III, 28029 Madrid, Spain; IdiSNA, Navarra Institute por Health Research, 31008 Barcelona, Navarra Spain

**Keywords:** Dietary adherence, Short-term dietary adherence, Long-term dietary adherence, Mediterranean Diet, PREDIMED trial, Dietary predictors, Dietary intervention

## Abstract

**Background:**

Dietary intervention success requires strong participant adherence, but very few studies have examined factors related to both short-term and long-term adherence. A better understanding of predictors of adherence is necessary to improve the design and execution of dietary intervention trials. This study was designed to identify participant characteristics at baseline and study features that predict short-term and long-term adherence with interventions promoting the Mediterranean-type diet (MedDiet) in the PREvención con DIeta MEDiterránea (PREDIMED) randomized trial.

**Methods:**

Analyses included men and women living in Spain aged 55–80 at high risk for cardiovascular disease. Participants were randomized to the MedDiet supplemented with either complementary extra-virgin olive oil (EVOO) or tree nuts. The control group and participants with insufficient information on adherence were excluded. PREDIMED began in 2003 and ended in 2010. Investigators assessed covariates at baseline and dietary information was updated yearly throughout follow-up. Adherence was measured with a validated 14-point Mediterranean-type diet adherence score. Logistic regression was used to examine associations between baseline characteristics and adherence at one and four years of follow-up.

**Results:**

Participants were randomized to the MedDiet supplemented with EVOO (*n* = 2,543; 1,962 after exclusions) or tree nuts (*n* = 2,454; 2,236 after exclusions). A higher number of cardiovascular risk factors, larger waist circumference, lower physical activity levels, lower total energy intake, poorer baseline adherence to the 14-point adherence score, and allocation to MedDiet + EVOO each independently predicted poorer adherence. Participants from PREDIMED recruiting centers with a higher total workload (measured as total number of persons-years of follow-up) achieved better adherence. No adverse events or side effects were reported.

**Conclusions:**

To maximize dietary adherence in dietary interventions, additional efforts to promote adherence should be used for participants with lower baseline adherence to the intended diet and poorer health status. The design of multicenter nutrition trials should prioritize few large centers with more participants in each, rather than many small centers.

**Trial registration:**

This study was registered at controlled-trials.com (http://www.controlled-trials. com/ISRCTN35739639). International Standard Randomized Controlled Trial Number (ISRCTN): 35739639. Registration date: 5 October 2005.

**Trial design:** parallel randomized trial.

**Electronic supplementary material:**

The online version of this article (doi:10.1186/s12966-016-0394-6) contains supplementary material, which is available to authorized users.

## Background

The global burden of diet-related chronic diseases has skyrocketed over the last few decades. As rates continue to rise [1] and food environments become increasingly obesogenic [2], it is more important than ever to improve understanding of diet-disease relationships and decrease the risk of chronic disease through diet modification. Effective dietary intervention strategies can help accomplish both of these objectives. The Mediterranean-type diet (MedDiet) is associated with decreased risk of all-cause [3, 4] and premature [5, 6] death, cardiovascular disease [7, 8], type 2 diabetes [9, 10], overweight and obesity [11, 12], and several cancers [13, 14]. Experimental interventions and randomized controlled trials (RCTs) have confirmed these findings [7, 8, 14–19]. It is very likely that future intervention trials in nutrition will adopt the paradigm of the Mediterranean food pattern, as the 2015 Dietary Guidelines for Americans have recommended [20]. In this context, information about the predictors of success in interventions using such a food pattern is very much needed.

Permanent dietary modifications are difficult to achieve; long-term dietary interventions often have low adherence [21–26]. Identifying participant characteristics and study design features that predict long-term adherence will substantially help investigators design dietary interventions to maximize adherence, achieve sufficient contrast in nutritional exposures between intervention arms, and reduce diet-related chronic diseases in target populations.

A handful of studies have identified predictors of short-term adherence to dietary interventions [27–35]. Predictors included the female sex [35], older age [27, 29], a non-diabetic [28, 35] and non-depressive status [34, 36], normal weight [29, 30, 34], higher physical activity levels [34, 35], not smoking [35], white ethnicity [29, 32], higher socioeconomic status [27, 29, 33], and being married [35]. Only two studies have investigated predictors of adherence to the MedDiet [35, 37]. PREvención con DIeta MEDiterránea (PREDIMED) researchers investigated the relationship between baseline characteristics and MedDiet adherence, but that study included only a partial sample of the trial and evaluated only short-term adherence [35]. Another study had longer follow-up but lacked a control group [37].

Among the very few studies that have examined long-term dietary adherence, the outcome was often defined as meeting weight-loss goals, which is only a proxy for dietary adherence. Because only sustained, long-term dietary patterns modify chronic disease risk [38–40], a better understanding of long-term adherence to healthy dietary patterns is needed.

The aim of the present study was to identify predictors of short and long-term adherence with a MedDiet intervention. This study uses data from the PREDIMED trial, a RCT of the MedDiet for the primary prevention of cardiovascular disease [41, 42].

## Methods

### Study population

Details on the PREDIMED design and methods are described in detail elsewhere [18, 23, 43]. Briefly, the PREDIMED trial was a multicenter, randomized, controlled, single-blinded cardiovascular disease prevention trial conducted in Spain [41]. It was designed to assess the effects of the MedDiet on cardiovascular disease in 7,447 participants recruited between 2003 and 2009. Eligible participants were aged 55 to 80 and at high risk for developing cardiovascular disease (CVD). High risk was defined as having type 2 diabetes or at least three of the following major (CVD) risk factors: current smoking, hypertension, elevated low-density lipoprotein cholesterol levels, BMI ≥25 kg/m^2^, or a family history of premature coronary heart disease (CHD). After providing written informed consent, participants were randomized to one of three interventions; a traditional MedDiet supplemented with either complementary extra-virgin olive oil (EVOO) or tree nuts or a control diet (advice to reduce all types of dietary fat). The control group was excluded from the present analyses because the focus of this study was the adherence with the intervention promoting the MedDiet. Inclusion criteria into this study are depicted in the flow diagram figure provided below, along with the CONSORT checklist for randomized trials. The trial ended in 2010, after a median follow-up of 4.8 years, because of the observed benefit of the MedDiet compared to the low-fat control diet in the prevention of CVD. Institutional Review Boards at 11 recruiting centers approved the study protocol [44]. No harm or unintended effects were reported in any arm [41].

When one-year dietary adherence was the outcome of interest, the present study excluded participants missing information on any of the 14-point dietary adherence score items at one year of follow-up (*n* = 799), leaving 4,198 participants for analysis. When four-year dietary adherence was the outcome of interest, participants recruited after November 2006 (*n* = 1,495) were excluded because subsequent follow-up was less than four years. Participants who had missing information on any of the 14-point dietary adherence score items at four years of follow-up were further excluded (*n* = 1,149), leaving 2,353 participants available for analysis.

### Outcome assessment

Registered dietitians conducted quarterly group sessions and one-on-one in-person interviews to deliver a comprehensive motivational educational intervention aimed at modifying participant eating habits. Dietitians collected detailed dietary information at baseline and yearly thereafter during follow-up. Individual interviews and group sessions were conducted every three months throughout the trial. A previously validated 14-item Mediterranean Diet Assessment Tool [45] was the primary method for assessing adherence to the intervention (Additional file 1: Figure S1). PREDIMED dietitians assessed participant adherence using this tool during each visit. A value of 0 (non-compliant) or 1 (compliant) was assigned to each item [46]. Higher scores reflected better adherence. High adherence was defined as meeting at least 11 of the 14 items. This cut-point was used because roughly half of participants complied with 11 or more items at each follow-up visit.

### Covariate assessment

Dietitians administered a validated 137-item food frequency questionnaire (FFQ) at each yearly visit [47], from which total energy and alcohol intake was computed [48]. Another questionnaire collected information on sociodemographic variables, lifestyle, and family history data. A validated Spanish version of the Minnesota questionnaire [49, 50] was used to assess physical activity. Investigators reviewed medical records at baseline and yearly thereafter to assess medical diagnoses. Nurses measured weight and height using standardized procedures, and blood pressure using a validated [51] semiautomatic oscillometer in triplicate (Omron HEM_705CP). Primary care doctors assessed participants for new diagnoses of hypercholesterolemia, hypertension and type 2 diabetes. Definitions for these diagnoses are described elsewhere [42].

### Exposure assessment

Potential baseline predictors of adherence were assessed based on clinical relevance and findings from previously published studies. These included sex (male, female), age (<65 years, ≥65 years), highest educational level attained (less than primary school, primary school, secondary school, university or more), occupation (retired, working, housewife, unemployed or unable to work), marital status (married, other), number of people in household, continuous cardiovascular risk factor score (one point was assigned for each of the following diagnoses or conditions: type 2 diabetes, hypertension, high blood cholesterol, family history of premature CHD, depression, and obesity; scores of 0–1 and 5–6 were collapsed due to low frequency), individual cardiovascular risk factors (each of the six factors included in the cardiovascular risk score were also assessed separately instead of as a continuous score), systolic blood pressure and diastolic blood pressure (SBP and DBP, continuous, per 5 mmHg), waist circumference (continuous, per 5 cm), physical activity (tertiles of MET-min/day), smoking status (never, former, current), total energy intake (quartiles of kcal/day), alcohol other than wine (low: <10 g/day for men, <5 for women; moderate: 10–50 for men, 5–10 for women; high: >50 for men, >10 for women), baseline 14-point dietary adherence score (<11 points, ≥11 points), and MedDiet intervention group (mixed nuts, EVOO). This analysis also evaluated whether the “total workload”, measured in person-years of follow-up and an indicator of how many participants a given center delivers the intervention to throughout follow-up, was associated with dietary adherence.

### Statistical methods

Chi-square tests were used to assess differences in distributions of baseline characteristics between those with low adherence (<11 points) and high adherence (≥11 points). In Table 2, crude and multivariate-adjusted logistic regression was used to calculate odds ratios (ORs) of adherence to the MedDiet at one and four years of follow-up according to baseline characteristics. Multivariate models were adjusted for all potential predictors of dietary adherence listed above. To calculate p-values for trend, the median value was assigned to each category and the resulting variable was treated as continuous. Quantiles were not treated as ordinal variables to account for the fact that the differences in median values across quantiles were not equal.

Several sensitivity analyses were conducted using multivariate logistic regression. Analyses looked at associations between potential baseline predictors and adherence at two and three years of follow-up (instead of one and four years), and with ≥10 and ≥12 as alternative cut-points for dietary adherence (instead of ≥11).

All p-values are two-tailed. Values of ≤0.05 are considered statistically significant. All statistical analyses were performed using Stata software (version 12.0, StataCorp 2011, College Station, TX, USA).

## Results

Table 1 shows the mean (±SD) or percentage of participants with high adherence and low adherence (≥11 and <11 points on 14-point score) after 1-y and 4-y follow-up across levels of baseline characteristics. 54 % of participants complied at one-year follow-up, and 58 % at 4-y follow-up. The mean (±SD) age at baseline was 66.9 (±6.1) years, and 56.4 % of participants were female. The following baseline characteristics were associated with lower adherence at both time points: female sex, a greater number of cardiovascular risk factors, larger waist circumference, less physical activity, less total energy intake, and lower baseline 14-point dietary adherence score. When individual risk factors were assessed, type 2 diabetes diagnosis and obesity were associated with poorer adherence at both time points. Randomization to the MedDiet supplemented with MedDiet + EVOO and inclusion at a center following a lower intervention workload were also correlated with lower adherence at one and four years. Additional file 2: Table S5 shows that distributions of baseline characteristics did not differ depending on time period of enrollment, with the exception of number of total workload per center. Centers that began recruitment after November, 2006 were the only centers with less than 300 participants. November, 2006 was selected as the cut-point because participants recruited after this date were followed for less than four years, and thus were excluded from analyses where dietary adherence at four years is the outcome of interest.

**Table 1:** Baseline characteristics according to a 14-point dietary adherence score after 1, 4 years of follow-up^a^

	1 Year of Follow-up			4 Years of Follow-up		
Adherence^b^	Low (*n* = 1925)	High (*n* = 2273)		Low (*n* = 978)	High (*n* = 1375)	
Demographic Characteristics ^c^	% or mean (SD)		*p*-value	% or mean (SD)		*p*-value
Women	59.1	54.6	0.003	61.3	54.6	0.001
Age at Baseline (years)	66.9 (6.1)	66.9 (5.9)	0.68	67.6 (6.2)	66.8 (5.8)	0.005
Educational level						
University	7.5	8.8		5.6	8.7	
Secondary school	14.8	16.2		15.8	15.0	
Primary school	74.7	72.8		75.0	74.8	
Less than primary school	3.1	2.2	0.06	3.6	1.6	0.001
Occupation						
Retired	51.6	54.6		50.9	53.0	
Working	13.1	11.3		10.4	11.4	
Housewife	31.5	31.6		35.7	33.0	
Unemployed/unable to work	3.8	2.6	0.02	3.0	2.7	0.51
Marital Status						
Married	75.9	78.9		75.6	78.9	
Single	3.7	3.7		3.5	3.9	
Widowed	18.6	15.1		19.1	15.4	
Divorced or separated	1.8	2.2	0.22	1.8	1.8	0.12
Number of People in Household	1.7 (1.3)	1.7 (1.4)	0.89	1.6 (1.1)	1.6 (1.5)	0.87
Health-Related Characteristics at Baseline						
Number of CVD Risk Factors^c^						
0-1	5.8	7.6		6.7	10.1	
2	27.1	31.5		28.6	33.0	
3	39.1	38.7		37.5	37.6	
4	21.9	17.7		21.2	16.2	
5-6	6.1	4.5	<0.001	6.0	3.1	<0.001
Type 2 diabetes	51.5	44.3	<0.001	53.1	44.6	<0.001
Hypertension	82.2	81.	0.60	82.2	80.2	0.21
Hypercholesterolemia	70.8	73.3	0.06	66.5	69.5	0.12
Family history of premature CHD	24.1	21.9	0.10	24.7	17.5	<0.001
Depression	18.1	16.2	0.10	17.9	14.8	0.04
Obesity	49.0	42.8	<0.001	47.4	42.7	0.02
SBP (mmHg)	148.5 (20.7)	149.7 (20.6)	0.07	148.5 (20.6)	150.5 (20.9)	0.02
DBP (mmHg)	82.9 (10.8)	83.5 (11.1)	0.09	83.3 (11.2)	84.1 (10.9)	0.10
Waist circumference (cm)	101.2 (10.0)	99.1 (10.6)	0.004	100.8 (10.3)	98.7 (10.0)	<0.001
Physical activity (MET-min/d)^d^						
T1 (low)	36.1	27.7		36.7	24.7	
T2	34.3	33.6		34.0	33.5	
T3 (high)	29.6	38.6	<0.001	29.4	41.8	<0.001
Smoking status						
Never	62.1	60.7		65.0	61.5	
Former	14.7	12.9		12.9	14.1	
Current	23.2	26.4	<0.03	22.1	24.4	0.22
Total energy intake (kcal/day)^e^						
Q1 (low)	27.3	21.4		24.6	19.9	
Q2	24.5	25.1		24.2	22.9	
Q3	25.3	26.5		23.7	27.0	
Q4 (high)	23.0	27.1	<0.001	27.6	30.3	0.02
Alcohol other than wine (g/day)						
< 10 men, <5 women	57.9	57.6		58.6	57.0	
10-50 men, 5–10 women	14.2	14.7		14.7	14.7	
> 50 men, > 10 women	27.9	27.7	0.90	26.7	28.3	0.68
14-point adherence score^b^	8.2 (1.8)	9.3 (1.8)	<0.001	8.5 (2.0)	9.3 (1.9)	<0.001
Intervention Design Features						
Intervention Group						
MedDiet + EVOO	58.0	49.2		60.7	53.2	
MedDiet + Nuts	42.0	50.8	<0.001	39.3	46.8	<0.001
Total workload of center (person-years) ^f^						
Q1 (low)	34.4	26.7		25.4	23.3	
Q2	27.3	26.8		41.3	33.0	
Q3	22.7	32.2		19.9	12.2	
Q4 (high)	15.6	15.4	<0.001	13.4	31.5	<0.001

^a^ Those randomized after November 2006 did not have the opportunity to provide information on 4-year adherence. ^b^ A validated MedDiet adherence assessment tool was used. 1 point was added for each item in adherence with the traditional MedDiet. High adherence = adherence with ≥11 items on 14-point dietary adherence score. Low adherence = adherence with <11 items. ^c^ Total CVD risk score was calculated by summing the following CVD risk factors: type 2 diabetes, hypertension, high blood cholesterol, family history of premature CHD, depression, obesity. ^d^ Tertiles of physical activity (MET-min/d): T1: <108; T2: 108–268; T3: ≥268. ^e^ Quartiles of energy intake (kcal/d), by sex: Men: Q1: <2051; Q2: 2051- < 2394; Q3:2934- < 2801; Q4: ≥2801. Women: Q1: <1786; Q2: 1786- < 2109; Q3: 2109- < 2465; Q4: ≥2465. ^f^ Measured in quartiles of person years at center. After 1 Year: Q1: 133- < 352; Q2: 352- < 537; Q3: 537- < 650; Q4: ≥650. After 4 years: Q1: 893- < 1220; Q2: 1220- < 2175; Q3: 2175- < 2384; Q4: ≥2384

**Fig. 1:**
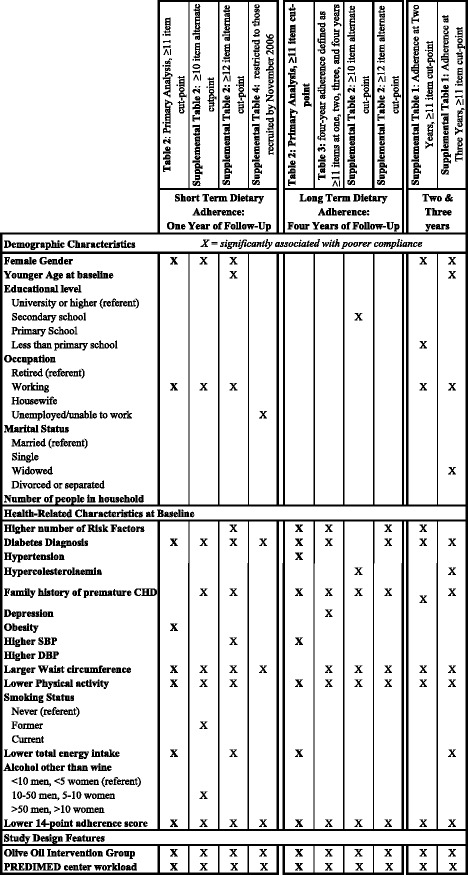
Summary of primary and sensitivity analysis results from multivariate logistic regression models investigating predictors of dietary adherence with the 14-point MedDiet score

Figure 1 provides a summary diagram comparing multivariate logistic regression results across all primary and sensitivity analyses.

### Short term dietary adherence (one year of follow-up)

Table 2 shows primary results for the association between potential baseline characteristics and dietary adherence after one and and four years of follow-up. The following baseline characteristics were associated with *lower* dietary adherence at one year of follow-up in multivariate logistic regression models: the female sex, working (vs. retired), type 2 diabetes diagnosis, obesity, larger waist circumference, lower physical activity, lower total energy intake, and lower 14-point baseline adherence score. Both study design features, randomization to the MedDiet + EVOO intervention arm, and belonging to a PREDIMED center that had a lower workload (fewer person years), were also associated with lower one-year adherence. In Additional file 2: Table S2, the dietary adherence score cut-off point is changed from ≥11 to the alternative cut-off points of ≥10 and ≥12. The majority of predictors of low one-year adherence observed in Table 2, when ≥11 items was the cut-off point, remained after changing the cut-off point. Exceptions include obesity (no longer a predictor when either alternate cut-off point was used) and lower energy intake (no longer a predictor when ≥10 items was the cut-off point). Additional file 2: Table S4 investigates adherence at one year of follow-up after excluding those recruited after November 2006 in order to restrict to the group that could be analyzed at both time points. A handful of associations did not hold, likely due to reduced power as a result of smaller sample size. Associations with lower one-year adherence that did not hold included the female gender, working (vs. retired), obesity, lower physical activity, and lower total energy intake. Baseline predictors of lower one-year adherence that remained significant throughout all sensitivity analyses include type 2 diabetes, larger waist circumference, and lower 14-point baseline adherence score. Both study design features (randomization to the MedDiet + EVOO intervention arm and belonging to a PREDIMED center with a lower workload) were also associated with lower one-year adherence throughout all sensitivity analyses.

**Table 2:** Odds of high adherence with the MedDiet intervention at one and four years of follow-up^a^

	OR (95 % CI) for dietary adherence (≥11 vs. <11 points)^b^									
	1 Year					4 Years				
Demographic Characteristics	n	Crude	*p*	Multivariate	*p*	n	Crude	*p*	Multivariate	*p*
Sex										
Men	1820	1.00 (ref)		1.00 (ref)		1103	1.00 (ref)		1.00 (ref)	
Women	2378	0.83 (0.73, 0.94)	0.003	0.78 (0.64, 0.96)	0.02	1350	0.76 (0.64, 0.90)	0.001	0.92 (0.69, 1.23)	0.59
Age at baseline (years)										
< 65	1627	1.00 (ref)		1.00 (ref)		868	1.00 (ref)		1.00 (ref)	
≥ 65	2571	1.01 (0.89, 1.14)	0.90	0.98 (0.80, 1.15)	0.80	1485	0.87 (0.74, 1.04)	0.12	0.90 (0.73, 1.12)	0.36
Educational level										
University or higher	339	1.00 (ref)		1.00 (ref)		173	1.00 (ref)		1.00 (ref)	
Secondary school	643	0.92 (0.71, 1.20)	0.56	0.97 (0.73, 1.28)	0.83	356	0.61 (0.42, 0.90)	0.01	0.68 (0.46, 1.02)	0.06
Primary School	3109	0.82 (0.65, 1.03)	0.09	0.90 (0.70, 1.15)	0.39	1768	0.64 (0.46, 0.90)	0.009	0.81 (0.57, 1.17)	0.26
Less than primary school	107	0.60 (0.39, 0.92)	0.02	0.87 (0.54, 1.39)	0.55	57	0.29 (0.15, 0.54)	<0.001	0.52 (0.27, 1.01)	0.06
Occupation										
Retired	2234	1.00 (ref)		1.00 (ref)		1127	1.00 (ref)		1.00 (ref)	
Working	509	0.81 (0.67, 0.98)	0.03	0.77 (0.61, 0.96)	0.02	258	1.04 (0.89, 1.37)	0.75	1.00 (0.72, 1.38)	0.99
Housewife	1324	0.95 (0.83, 1.09)	0.44	1.12 (0.93, 1.33)	0.51	802	0.89 (0.74, 1.06)	0.19	1.03 (0.81, 1.32)	0.80
Unemployed/unable to work	131	0.64 (0.45, 0.91)	0.01	0.14 (0.47, 1.01)	0.06	66	0.87 (0.53, 1.44)	0.59	1.00 (0.58, 1.73)	0.99
Marital Status										
Married	3266	1.00 (ref)		1.00 (ref)		1824	1.00 (ref)		1.00 (ref)	
Single	157	0.97 (0.70, 1.34)	0.85	1.02 (0.72, 1.43)	0.92	88	1.08 (0.70, 1.68)	0.73	0.93 (0.58, 1.49)	0.76
Widowed	668	0.87 (0.74, 1.03)	0.07	1.00 (0.83, 1.21)	0.99	399	0.77 (0.62, 0.96)	0.02	0.86 (0.67, 1.11)	0.26
Divorced or separated	107	0.75 (0.51, 1.10)	0.15	0.99 (0.66, 1.50)	0.97	42	0.91 (.49, 1.69)	0.76	1.16 (0.60, 2.25)	0.65
Number of people in household	4198	1.00 (0.96, 1.05)	0.89	1.04 (0.99, 1.10)	0.12	2353	1.01 (0.94, 1.07)	0.87	0.99 (0.92, 1.06)	0.70
Health-Related Characteristics at Baseline										
Number of CVD Risk Factors ^c,d^										
0–1	285	1.00 (ref)		1.00 (ref)		204	1.00 (ref)		1.00 (ref)	
2	1237	0.89 (0.68, 1.15)		0.94 (0.71, 1.25)		734	0.76 (0.55, 1.05)		0.82 (0.58, 1.17)	
3	1632	0.76 (0.59, 0.98)		0.94 (0.71, 1.24)		884	0.66 (0.48, 0.91)		0.83 (0.58, 1.18)	
4	824	0.62 (0.47, 0.81)		0.87 (0.64, 1.18)		429	0.50 (0.35, 0.71)		0.71 (0.48, 1.04)	
5–6	220	0.57 (0.40, 0.81)	<0.001	0.83 (0.56, 1.23)	0.26	102	0.34 (0.21, 0.56)	<0.001	0.47 (0.27, 0.80)	0.009
Individual CVD Risk Factors										
Type 2 Diabetes										
No	2199	1.00 (ref)		1.00 (ref)		1221	1.00 (ref)		1.00 (ref)	
Yes	1999	0.75 (0.66, 0.85)	<0.001	0.77 (0.66, 0.88)	<0.001	1132	0.71 (0.60, 0.84)	<0.001	0.74 (0.61, 0.90)	0.003
Hypertension										
No	757	1.00 (ref)		1.00 (ref)		447	1.00 (ref)		1.00 (ref)	
Yes	3441	0.97 (0.83, 1.13)	0.68	0.89 (0.74, 1.06)	0.20	1906	0.87 (0.71, 1.08)	0.21	0.77 (0.61, 0.99)	0.04
Hypercolesterolaemia										
No	1169	1.00 (ref)		1.00 (ref)		748	1.00 (ref)		1.00 (ref)	
Yes	3029	1.14 (0.99, 1.30)	0.06	1.06 (0.92, 1.24)	0.42	1605	1.15 (0.96, 1.37)	0.13	1.11 (0.91, 1.36)	0.29
Family history of premature CHD										
No	3237	1.00 (ref)		1.00 (ref)		1871	1.00 (ref)		1.00 (ref)	
Yes	961	0.89 (0.77, 1.02)	0.10	0.88 (0.75, 1.03)	0.11	482	0.64 (0.53, 0.79)	<0.001	0.63 (0.50, 0.78)	<0.001
Depression										
No	3481	1.00 (ref)		1.00 (ref)		1975	1.00 (ref)		1.00 (ref)	
Yes	717	0.87 (0.74, 1.02)	0.10	0.97 (0.81, 1.15)	0.71	378	0.79 (0.64, 0.99)	0.04	0.81 (0.63, 1.03)	0.09
Obesity										
No	2282	1.00 (ref)		1.00 (ref)		1302	1.00 (ref)		1.00 (ref)	
Yes	1916	0.78 (0.69, 0.88)	<0.001	1.12 (0.95, 1.32)	0.17	1051	0.83 (0.70, 0.97)	0.02	1.14 (0.91, 1.43)	0.24
SBP (per 5 mmHg)	4198	1.01 (1.00, 1.03)	0.07	1.00 (0.99, 1.02)	0.70	2353	1.02 (1.00, 1.04)	0.02	1.01 (0.99, 1.04)	0.31
DBP (per 5 mmHg)	4198	1.02 (1.00, 1.05)	0.09	1.02 (0.98, 1.05)	0.38	2353	1.03 (0.99, 1.07)	0.10	1.00 (0.95, 1.05)	0.90
Waist circumference (per 5 cm)	4198	0.90 (0.88, 0.93)	<0.001	0.92 (0.88, 0.95)	<0.001	2353	0.90 (0.87, 0.94)	<0.001	0.97 (0.92, 1.02)	0.22
Physical activity (MET-min/d) ^c,e^										
T1 (low)	1332	1.00 (ref)		1.00 (ref)		703	1.00 (ref)		1.00 (ref)	
T2	1414	1.28 (1.10, 1.48)		1.13 (0.97, 1.33)		785	1.46 (1.19, 1.80)		1.29 (1.04, 1.60)	
T3 (high)	1452	1.70 (1.46, 1.98)	<0.001	1.38 (1.17, 1.63)	<0.001	865	2.12 (1.72, 2.60)	<0.001	1.62 (1.29, 2.04)	<0.001
Smoking Status										
Never	2576	1.00 (ref)		1.00 (ref)		1482	1.00 (ref)		1.00 (ref)	
Former	574	0.90 (0.75, 1.08)	0.24	0.83 (0.67, 1.04)	0.10	320	1.16 (0.90, 1.48)	0.25	0.97 (0.71, 1.31)	0.83
Current	1048	1.17 (1.01, 1.35)	0.04	1.05 (0.87, 1.26)	0.63	551	1.17 (0.95, 1.42)	0.13	1.01 (0.78, 1.30)	0.96
Total energy intake (kcal/day) ^c, f^										
Q1 (low)	1017	1.00 (ref)		1.00 (ref)		568	1.00 (ref)		1.00 (ref)	
Q2	1038	1.31 (1.10, 1.56)		1.20 (1.00, 1.45)		858	1.17 (0.92, 1.49)		1.08 (0.84, 1.40)	
Q3	1078	1.34 (1.13, 1.59)		1.16 (0.96, 1.39)		363	1.41 (1.11, 1.79)		1.28 (0.99, 1.65)	
Q4 (high)	1065	1.50 (1.26, 1.79)	<0.001	1.32 (1.09, 1.58)	0.009	564	1.36 (1.08, 1.72)	0.007	1.27 (0.99, 1.64)	0.04
Alcohol other than wine (g/day)										
< 10 men, <5 women	2429	1.00 (ref)		1.00 (ref)		1360	1.00 (ref)		1.00 (ref)	
10-50 men, 5–10 women	606	1.04 (0.87, 1.24)	0.67	0.88 (0.72, 1.08)	0.21	344	1.03 (0.81, 1.31)	0.81	0.80 (0.61, 1.05)	0.11
> 50 men, >10 women	1163	1.00 (0.87, 1.15)	0.97	0.93 (0.80, 1.09)	0.39	649	1.09 (0.90, 1.32)	0.38	1.03 (0.83, 1.28)	0.77
14-point adherence score ^a^										
< 11	3416	1.00 (ref)		1.00 (ref)		1864	1.00 (ref)		1.00 (ref)	
≥ 11	782	3.41 (2.85, 4.07)	<0.001	3.25 (2.71, 3.91)	<0.001	489	2.06 (1.66, 2.6)	<0.001	1.81 (1.44, 2.27)	<0.001
Study Design Features										
Intervention Group										
MedDiet + nuts	1962	1.00 (ref)		1.00 (ref)		1027	1.00 (ref)		1.00 (ref)	
MedDiet + EVOO	2236	0.70 (0.62, 0.79)	<0.001	0.70 (0.62, 0.80)	<0.001	1326	0.74 (0.62, 0.87)	<0.001	0.74 (0.62, 0.88)	0.001
Total workload of center (person-years) ^e,g^										
Q1 (low)	1247	1.00 (ref)		1.00 (ref)		624	1.00 (ref)		1.00 (ref)	
Q2	1133	1.31 (1.12, 1.54)		1.34 (1.12, 1.59)		361	0.87 (0.70, 1.08)		0.87 (0.69, 1.10)	
Q3	1168	1.90 (1.61, 2.23)		1.75 (1.46, 2.10)		804	0.67 (0.51, 0.87)		0.74 (0.56, 0.99)	
Q4 (high)	650	1.32 (1.10, 1.60)	<0.001	1.48 (1.19, 1.79)	<0.001	564	2.56 (1.98, 3.31)	<0.001	2.27 (1.72, 3.00)	0.004

^a^ ORs < 1 imply poorer adherence. ORs > 1 imply better adherence. A validated MedDiet adherence assessment tool was used. 1 point was added for each item in adherence with the traditional MedDiet. High adherence = adherence with ≥11 items on 14-point dietary adherence score. Low adherence = adherence with <11 items. ^b^ All models are from logistic regression analysis. Multivariate models are mutually adjusted for all characteristics displayed in this table including total CVD risk score but excluding individual CVD risk factors (type 2 diabetes, hypertension, hypercholesterolaemia, family history of pre-mature CHD, depression, obesity). When an individual CVD risk factors was the exposure of interest, the model was mutually adjusted for other individual CVD risk factors but not total CVD risk score. ^c^ P-values for trend were calculated by assigning the median value to each category and treating the resulting variable as continuous ^d^ Total CVD risk score calculated by summing the following CVD risk factors: type 2 diabetes, hypertension, high blood cholesterol, family history of premature CHD, depression, obesity. ^e^ Tertiles of physical activity (MET-min/d): T1: <108; T2: 108–268; T3: ≥268. ^f^ Quartiles of energy intake (kcal/d), by sex: Men: Q1: <2051; Q2: 2051- < 2394; Q3:2934- < 2801; Q4: ≥2801. Women: Q1: <1786; Q2: 1786- < 2109; Q3: 2109- < 2465; Q4: ≥2465. ^g^ Measured in quartiles of person years at center. After 1 Year: Q1: 133- < 352; Q2: 352- < 537; Q3: 537- < 650; Q4: ≥650. After 4 years: Q1: 893- < 1220; Q2: 1220- < 2175; Q3: 2175- < 2384; Q4: ≥2384

### Long term dietary adherence (after four years of follow-up)

Based on the primary analysis in Table 2, the following baseline characteristics were associated with lower dietary adherence after four years in multivariate logistic regression models: higher total number of cardiovascular risk factors, specifically type 2 diabetes diagnosis, hypertension, and family history of premature CHD, higher SBP, lower physical activity levels, lower total energy intake, and lower baseline14-point adherence score. Study design features predicting lower adherence after four years of follow-up included being in the MedDiet + EVOO intervention arm and belonging to a PREDIMED center with a lower workload over follow-up. Table 3 defines four-year adherence as consistently meeting the criteria for high dietary adherence (≥11 points on 14-point score) every year throughout the first four years of follow-up. Results were similar to four-year results in Table 2. However, with this more stringent definition, hypertension, higher SBP, and lower energy intake were no longer associated with poorer four-year adherence. After changing the dietary adherence cut-off points to ≥10 and ≥12 items (Additional file 2: Table S2), all associations between potential predictors and lower four-year adherence remained except for total number of cardiovascular risk factors (no longer a predictor when cut-off point was ≥10 items), type 2 diabetes diagnosis (no longer a predictor when cut-point was ≥10 items), higher SBP (no longer a predictor for either alternative cut-point), and lower total energy intake (no longer a predictor using either alternative cut-off point). Baseline predictors of lower four-year adherence that remained significant throughout all sensitivity analyses included family history of CHD, lower physical activity, lower baseline 14-point adherence score, randomization to the MedDiet + EVOO arm, and belonging to a PREDIMED center with a lower workload.

**Table 3:** Odds of high adherence with the MedDiet intervention every year throughout four years of follow-up. ^a^

	OR (95 % CI) for dietary adherence (≥11 vs. <11 points)^b^				
Demographic Characteristics	n	Crude Model	*p*	Multivariate	*p*
Sex					
Men	815	1.00 (ref)		1.00 (ref)	
Women	1103	0.69 (0.57, 0.84)	<0.001	0.72 (0.49, 1.05)	0.09
Age at baseline (years)					
< 65	683	1.00 (ref)		1.00 (ref)	
≥ 65	1235	0.94 (0.77, 1.15)	0.57	0.85 (0.65, 1.12)	0.25
Educational level					
University or higher	146	1.00 (ref)		1.00 (ref)	
Secondary school	294	0.59 (0.39, 0.89)	0.01	0.71 (0.44, 1.14)	0.15
Primary School	1435	0.62 (0.44, 0.89)	0.006	0.78 (0.52, 1.18)	0.24
Less than primary school	43	0.23 (0.09, 0.57)	0.002	0.53 (0.19, 1.48)	0.23
Occupation					
Retired	1017	1.00 (ref)		1.00 (ref)	
Working	197	0.79 (0.56, 1.10)	0.16	0.72 (0.47, 1.11)	0.14
Housewife	662	0.85 (0.68, 1.05)	0.12	1.00 (0.72, 1.38)	0.98
Unemployed/unable to work	42	0.48 (0.22, 1.04)	0.06	0.72 (0.30, 1.71)	0.46
Marital Status					
Married	1484	1.00 (ref)		1.00 (ref)	
Single	76	1.04 (0.64, 1.71)	0.86	0.85 (0.47, 1.51)	0.58
Widowed	324	0.78 (0.60, 1.02)	0.07	0.89 (0.64, 1.24)	0.50
Divorced or separated	34	0.66 (0.29, 1.46)	0.30	1.35 (0.54, 3.40)	0.52
Number of people in household	1918	1.05 (0.96, 1.14)	0.31	1.02 (0.91, 1.14)	0.76
Health-Related Characteristics at Baseline					
Number of CVD Risk Factors ^c,d^					
0–1	171	1.00 (ref)		1.00 (ref)	
2	595	0.95 (0.67, 1.35)		1.13 (0.75, 1.68)	
3	714	0.62 (0.44, 1.87)		0.91 (0.60, 1.37)	
4	352	0.45 (0.30, 0.66)		0.78 (0.48, 1.25)	
5–6	86	0.18 (0.08, 0.38)	<0.001	0.27 (0.12, 0.63)	0.003
Type 2 Diabetes					
No	993	1.00 (ref)		1.00 (ref)	
Yes	925	0.63 (0.52, 0.77)	<0.001	0.73 (0.56, 0.93)	0.01
Hypertension					
No	367	1.00 (ref)		1.00 (ref)	
Yes	1551	0.89 (0.70, 1.14)	0.36	0.75 (0.56, 1.02)	0.07
Hypercolesterolaemia					
No	604	1.00 (ref)		1.00 (ref)	
Yes	1314	1.04 (0.84, 1.28)	0.75	1.12 (0.87, 1.44)	0.39
Family history of premature CHD					
No	1537	1.00 (ref)		1.00 (ref)	
Yes	381	0.74 (0.57, 0.95)	0.02	0.72 (0.54, 0.97)	0.03
Depression					
No	1607	1.00 (ref)		1.00 (ref)	
Yes	311	0.72 (0.55, 0.95)	0.02	0.69 (0.50, 0.95)	0.02
Obesity					
No	1054	1.00 (ref)		1.00 (ref)	
Yes	864	0.57 (0.47, 0.70)	<0.001	0.84 (0.63, 1.12)	0.23
SBP (per 5 mmHg)	1918	1.03 (1.01, 1.06)	0.004	1.02 (0.98, 1.05)	0.34
DBP (per 5 mmHg)	1918	1.04 (1.00, 1.09)	0.04	1.00 (0.94, 1.07)	0.94
Waist circumference (per 5 cm)	1918	0.82 (0.78, 0.87)	<0.001	0.93 (0.88, 1.00)	0.04
Physical activity (MET-min/d) ^c,e^					
T1 (low)	552	1.00 (ref)		1.00 (ref)	
T2	642	1.62 (1.24, 2.12)		1.27 (0.94, 1.71)	
T3 (high)	724	2.63 (2.04, 3.40)	<0.001	1.60 (0.18, 2.17)	0.002
Smoking Status					
Never	1209	1.00 (ref)		1.00 (ref)	
Former	251	1.04 (0.78, 1.41)	0.75	0.75 (0.51, 1.11)	0.15
Current	458	1.08 (0.86, 1.36)	0.51	0.82 (0.59, 1.14)	0.25
Total energy intake (kcal/day) ^c,f^					
Q1 (low)	405	1.00 (ref)		1.00 (ref)	
Q2	443	1.11 (0.82, 1.50)		1.00 (0.71, 1.40)	
Q3	499	1.37 (1.03, 1.82)		1.19 (0.84, 1.65)	
Q4 (high)	571	1.15 (0.87, 1.53)	0.29	1.12 (0.80, 1.56)	0.39
Alcohol other than wine (g/day)					
< 10 men, <5 women	1125	1.00 (ref)		1.00 (ref)	
10-50 men, 5–10 women	270	1.25 (0.95, 1.65)	0.12	0.87 (0.61, 1.23)	0.43
> 50 men, >10 women	523	0.95 (0.75, 1.19)	0.63	0.95 (0.72, 1.25)	0.69
14-point adherence score ^a^					
< 11	1528	1.00 (ref)		1.00 (ref)	
≥ 11	390	2.95 (2.34, 3.71)	<0.001	2.63 (2.02, 3.42)	<0.001
Study Design Features					
Intervention Group					
MedDiet + Nuts	809	1.00 (ref)		1.00 (ref)	
MedDiet + EVOO	1109	0.68 (0.56, 0.82)	<0.001	0.66 (0.53, 0.83)	<0.001
Total workload of center (person-years) ^e,g^					
Q1 (low)	267	1.00 (ref)		1.00 (ref)	
Q2	809	0.54 (0.39, 0.74)		0.56 (0.40, 0.80)	
Q3	311	0.45 (0.30, 0.67)		0.52 (0.34, 0.80)	
Q4 (high)	531	3.50 (2.55, 4.79)	<0.001	3.17 (2.22, 4.52)	<0.001

^a^ ORs < 1 imply poorer adherence. ORs > 1 imply better adherence. A validated MedDiet adherence assessment tool was used. 1 point was added for each item in adherence with the traditional MedDiet. High adherence = adherence with ≥11 items on 14-point dietary adherence score. Low adherence = adherence with <11 items. ^b^ All models are from logistic regression analysis. Multivariate models are mutually adjusted for all characteristics displayed in this table including total CVD risk score but excluding individual CVD risk factors (type 2 diabetes, hypertension, hypercholesterolaemia, family history of pre-mature CHD, depression, obesity). When an individual CVD risk factors was the exposure of interest, the model was mutually adjusted for other individual CVD risk factors but not total CVD risk score. ^c^ P-values for trend were calculated by assigning the median value to each category and treating the resulting variable as continuous. ^d^ Risk score calculated by summing the following CVD risk factors: type 2 diabetes, hypertension, high blood cholesterol, family history of premature CHD, depression, obesity. ^e^ Tertiles of physical activity (MET-min/d): T1: <108; T2: 108–268; T3: ≥268. ^f^ Quartiles of energy intake (kcal/d), by sex: Men: Q1: <2051; Q2: 2051- < 2394; Q3:2934- < 2801; Q4: ≥2801. Women: Q1: <1786; Q2: 1786- < 2109; Q3: 2109- < 2465; Q4: ≥2465. ^g^ Measured in quartiles of person years at center. After 4 years: Q1: 893- < 1220; Q2: 1220- < 2175; Q3: 2175- < 2384; Q4: ≥2384

### Medium-term adherence (two and three years of follow-up)

Additional file 2: Table S1 shows results for the association between potential predictors and adherence at the alternate time points of two and three years of follow-up. All characteristics that predicted lower adherence at both one and four years in the primary analysis multivariate logistic regression models (type 2 diabetes diagnosis, lower physical activity, lower total energy intake, lower 14-point adherence score, randomization to the MedDiet + EVOO arm, and belonging to a PREDIMED center with a lower workload) also predicted low adherence at both two and three years.

Additional file 2: Table S3 presents results from logistic regression analyses of the association between MedDiet intervention (nuts or EVOO) and dietary adherence to nut and olive oil items on the 14-point dietary adherence score (≥4 tbsp olive oil per day; olive oil as main culinary fat; ≥3 servings of nuts per week). Those in the MedDiet + EVOO intervention arm had significantly higher odds of complying with either of the two olive oil items (5 to 10 times the odds) at both one and four years of follow-up. In contrast, those in the nut intervention group had about 20 times the odds of complying with the nut item.

## Discussion

In the PREDIMED trial, baseline characteristics showing the strongest associations with both low short-term and low long-term dietary adherence with a MedDiet intervention included a higher number of cardiovascular risk factors (including specifically type 2 diabetes diagnosis), larger waist circumference, lower levels of physical activity, lower baseline dietary adherence, randomization to the MedDiet + EVOO intervention arm and belonging to a PREDIMED center with a lower workload, measured by total person years of follow-up.

### Study design

It is not surprising that the total workload (measured in person years) at a PREDIMED center was associated with both short-term and long-term adherence; the workload likely represents the level of experience the research team had with intervention delivery. Similar findings have been observed in hospitals, where quality of care is often related to number of administered procedures [52]. This finding suggests that multicenter interventions should recruit participants to fewer centers with more participants in each, instead of more centers with fewer participants in each, to maximize effectiveness and adherence. Streamlining intervention delivery would have an added benefit of reducing costs. While this would not explain the difference in adherence, this would free up resources for increased support for participants at risk of poor or suboptimal adherence.

Participants randomized to the MedDiet + EVOO (compared to tree nuts) had lower dietary adherence. This is probably because olive oil is a staple ingredient in the Spanish diet; participants consume olive oil regardless of supplementation from PREDIMED. Nut consumption is not as commonplace. As a result, it is easier for the nut group to adhere to the olive oil criteria compared to the olive oil group’s ability to adhere to the nut criteria. Additional file 2: Table S3 shows that intervention group is a much stronger predictor of complying with the nut adherence item compared to the olive oil adherence items. This suggests that, for dietary interventions providing participants with complementary food items, it may be most effective to provide them with foods that are less commonplace.

### Baseline health and lifestyle characteristics

In the present study, many predictors of low adherence with the MedDiet are indicators of poorer baseline health, including various cardiovascular risk factors, less physical activity, and poorer baseline diet. These results are consistent with previous findings investigating predictors of adherence with dietary interventions for reducing fat [29] and carbohydrates [28], family-level interventions [34], and MedDiet interventions [35, 37]. Baseline health status may indicate how much a person values his or her health, which may moderate one’s motivation to comply with the intervention. Alternatively, some research suggests that individuals may be more willing or motivated to make dietary and lifestyle improvements following a medical diagnosis [53]. These findings do not necessarily contradict this notion, as many of these indicators of baseline health are likely long-standing conditions and/or habits; the time during which one is more motivated to make improvements may have passed. Regardless, unhealthy individuals have a greater need for dietary improvement. Thus, they are often the most important targets of dietary interventions. Personalized, higher-intensity intervention approaches may be needed to achieve optimal adherence among less healthy individuals.

### Demographic characteristics

Like this study, most previous studies [35, 37] found that women have lower adherence than men. The only exception was a family intervention study [34]. It is possible that because mothers traditionally plan family meals, they were motivated to set a positive example through intervention adherence [34]. However, in this study population, it is possible that spouses and children influence meal preparation, leading to these disparate findings. Different strategies likely have different levels of effectiveness based on sex [54]. However, in the present study the female sex only predicted lower adherence at one year of follow-up, and not four years.

There have been conflicting findings about the relationship between age and dietary adherence [27, 29, 37]. The age range in the present study was restricted to 55–80 years; hence little age variability likely limited the ability to detect an association.

There was little evidence for an association between educational attainment and intervention adherence. While participants with less than a primary school education had lower long-term adherence than those with university level or higher, this did not hold in several sensitivity analyses. Previous studies have found that higher socioeconomic status predicted better dietary adherence, but findings did not hold for long-term adherence [27, 29] and were limited to low-fat dietary interventions [33]. This suggests that dietary interventions may be able to overcome the socioeconomic disparities that often exist in nutrition [55].

### Further discussion

The present study has several strengths. First, the sample size was large and it was conducted in an established, long-term, and successful randomized trial. Second, because all study participants were at high risk for CVD, it was possible to assess adherence among participants who were less healthy compared to the general population. Because they also were likely to have poorer baseline diets, adherence was probably especially challenging for these individuals. Thus, significant predictors of adherence may be even more meaningful in this setting. Third, this is one of the few studies that has been able to assess long-term dietary adherence. This is critical, as long-term, high-quality dietary pattern is the relevant dietary exposure for the prevention of chronic disease. Fourth, mutually adjusting for a wide array of baseline characteristics minimized residual confounding. Lastly, significant measurement error is unlikely because only 0.3 % of covariate values were missing, a validated measure for assessing dietary adherence was used [46], and previous analyses show that self-reported dietary intake is highly correlated with biomarkers in this population [18, 23].

It is important to note that because the high adherence is not rare, the ORs do not approximate risk ratios (RRs) and thus should not be incorrectly interpreted as RRs. However, provided appropriate interpretation, ORs still provide valid estimates, and it is more appropriate to apply OR estimates to all individuals within a population. Furthermore, because an OR incorporates both success and failure symmetrically, it is less arbitrary than a RR and thus a more robust estimate [56].

There are also limitations in this study. The potential for measurement error always exists. To include as many people as possible in the present analyses, missing covariate values were imputed for 0.3 % of values. Recall bias, social desirability bias and differential misreporting are always possible when diet is self-reported. Finally, it is always possible that failure to control for unmeasured confounders may have distorted results for predictors of dietary adherence. However, analyses were adjusted for a wide array of important baseline characteristics, and a strong confounder unrelated to these characteristics is unlikely. This unique population of older Spanish participants at high risk for cardiovascular disease may have low generalizability to the general public at lower risk of CVD.

The relative success of a dietary intervention to induce changes in the overall food pattern has been more frequently ascribed to strategies related to negotiation, goal setting, self monitoring, and skill building [57–59]. Other strategies such as the training of dietitians, length and intensity of intervention, frequency of contacts, multiplicity of channels used for the delivery of the intervention, the initial motivation of participants for adherence, and the provision of appropriate means for feedback should not be forgotten.

It is clear that certain participants have greater difficulty complying with dietary interventions. Our results identify specific baseline characteristics that predict better adherence, which is an instrumental first step for designing personalized intervention delivery strategies. However, further research is needed to also identify barriers to dietary adherence. Identifying both individual and universal barriers will have important implications for exactly how to promote adherence, and allow for an even more targeted and personalized intervention approach.

## Conclusion

Investigators should design dietary interventions for maximum dietary adherence. Long-term adherence is especially important. With a growing worldwide interest in interventions promoting the MedDiet, these results suggest the need for an early identification of participants with lower baseline adherence to a healthy diet and poorer health status. Additional efforts to promote adherence might be required among this group. Further research is needed to identify the most effective approach for overcoming the inherent difficulties in achieving optimal adherence, including identifying barriers to dietary change and adherence at an individual level. For multi-centered studies, it may be more effective to streamline intervention delivery by allocating participants to few large centers rather to many small centers; a higher volume of participants per dietitian in these large center will be more effective to obtain adherence. Dietary intervention studies designed to maximize adherence will contribute higher quality public health research and generate more effective and permanent dietary improvements among participants. This will ultimately decrease the burden of diet-related non-communicable diseases.

## Abbreviations

CHD, coronary heart disease; CVD, cardiovascular disease; DBP, diastolic blood pressure; EVOO, extra virgin olive oil; FFQ, food frequency questionnaire; MedDiet, Mediterranean-type diet; MET, metabolic equivalent of task; OR, odds ratio; PREDIMED, PREvención con DIeta MEDiterránea; RCTs, randomized controlled trials; RR, risk ratio; SBP, systolic blood pressure; SD, standard deviation

## Additional files

Additional file 1:
**Table S1.** Odds of high adherence with the MedDiet intervention at two and three years of follow-up. **Table S2.** Odds of high adherence with the MedDiet intervention using alternate adherence score cut-points. **Table S3.** Odds of adherence with olive oil and nut consumption after 1 and 4 years of follow-up. **Table S4.** Odds of high adherence with the MedDiet intervention at one year^a^, restricting the analyses to those participants recruited before 2006. **Table S5.** Adherence at one year of follow-up according to a 14-point dietary adherence score and year of recruitment into PREDIMED. **Table S6.** Odds of high adherence with the MedDiet intervention at one and four years of follow-up^a^, with alternate representation of “total workload”. (DOC 615 kb) Additional file 2:
**Figure S1.** Validated 14-item questionnaire of mediterranean diet adherence (DOCX 205 kb) Additional file 3: Consort 2010 Checklist. (DOC 219 kb)
