# Supplementary material for: Predictors of short- and long-term adherence with a Mediterranean-type diet intervention: the PREDIMED randomized trial
Source: Int J Behav Nutr Phys Act. 2016 Jun 14;13:67. doi: 10.1186/s12966-016-0394-6 (PMC4907003; doi:10.1186/s12966-016-0394-6)
Supplement: Additional file 1: — Table S1. Odds of high adherence with the MedDiet intervention at two and three years of follow-up. Table S2. Odds of high adherence with the MedDiet intervention using alternate adherence score cut-points. Table S3. Odds of adherence with olive oil and nut consumption after 1 and 4 years of follow-up. Table S4. Odds of high adherence with the MedDiet intervention at one yeara, restricting the analyses to those participants recruited before 2006. Table S5. Adherence at one year of follow-up according to a 14-point dietary adherence score and year of recruitment into PREDIMED. Table S6. Odds of high adherence with the MedDiet intervention at one and four years of follow-upa, with alternate representation of “total workload”. (DOC 615 kb) [file 12966_2016_394_MOESM1_ESM.doc]

**Additional file 1: Table S1. Odds of high adherence with the MedDiet intervention at two and three years of follow-up^a^.**

|  | **Multivariate OR (95% CI) for dietary adherence**  **(≥11 vs. <11 points)^b^** | | | | | |
| --- | --- | --- | --- | --- | --- | --- |
| **Demographic Characteristics** | n | 2 years | *p* | n | 3 years | *p* |
| **Sex** |  |  |  |  |  |  |
| Men | 1642 | 1.00 (ref) |  | 1550 | 1.00 (ref) |  |
| Women | 2218 | 0.75 (0.61, 0.93) | 0.01 | 2094 | 0.71 (0.56, 0.89) | 0.003 |
| **Age at baseline** (years) |  |  |  |  |  |  |
| <65 | 1457 | 1.00 (ref) |  | 1411 | 1.00 (ref) |  |
| ≥65 | 2403 | 0.89 (0.76, 1.05) | 0.17 | 2233 | 0.84 (0.71, 1.00) | 0.05 |
| **Educational level** |  |  |  |  |  |  |
| University or higher | 305 | 1.00 (ref) |  | 271 | 1.00 (ref) |  |
| Secondary school | 583 | 1.15 (0.86, 1.55) | 0.35 | 562 | 0.94 (0.69, 1.29) | 0.70 |
| Primary School | 2866 | 0.94 (0.73, 1.23) | 0.67 | 2706 | 0.91 (0.68, 1.21) | 0.51 |
| Less than primary school | 106 | 0.63 (0.39 1.02) | 0.06 | 105 | 0.68 (0.41, 1.12) | 0.13 |
| **Occupation** |  |  |  |  |  |  |
| Retired | 2079 | 1.00 (ref) |  | 1903 | 1.00 (ref) |  |
| Working | 444 | 0.85 (0.59, 0.95) | 0.02 | 447 | 0.72 (0.56, 0.92) | 0.01 |
| Housewife | 1220 | 1.06 (0.88, 1.27) | 0.57 | 1178 | 1.09 (0.90, 1.32) | 0.40 |
| Unemployed/unable to work | 117 | 0.85 (0.56, 1.270 | 0.42 | 116 | 0.90 (0.59, 1.36) | 0.61 |
| **Marital Status** |  |  |  |  |  |  |
| Married | 2993 | 1.00 (ref) |  | 2818 | 1.00 (ref) |  |
| Single | 141 | 0.85 (0.59, 1.22) | 0.38 | 130 | 0.76 (0.52, 1.11) | 0.15 |
| Widowed | 631 | 0.86 (0.71, 1.05) | 0.14 | 611 | 0.78 (0.63, 0.95) | 0.02 |
| Divorced or separated | 95 | 0.80 (0.52, 1.23) | 0.31 | 85 | 0.65 (0.41, 1.04) | 0.07 |
| **Number of people in household** | 3860 | 1.04 (0.98, 1.10) | 0.18 | 3644 | 0.96 (0.90, 1.03) | 0.24 |
| **Health-Related Characteristics at Baseline** | | |  |  |  |  |
| **Number of CVD Risk Factors** ^c,d^ |  |  |  |  |  |  |
| 0-1 | 255 | 1.00 (ref) |  | 257 | 1.00 (ref) |  |
| 2 | 1127 | 1.01 (0.75, 1.36) |  | 1048 | 1.21 (0.90, 1.63) |  |
| 3 | 1499 | 0.93 (0.69, 1.25) |  | 1404 | 1.26 (0.93, 1.70) |  |
| 4 | 779 | 0.83 (0.60, 1.15) |  | 739 | 1.18 (0.85, 1.63) |  |
| 5-6 | 200 | 0.74 (0.49, 1.12) | 0.03 | 196 | 0.97 (0.64, 1.47) | 0.87 |
| **Type 2 Diabetes** |  |  |  |  |  |  |
| No | 2008 | 1.00 (ref) |  | 1874 | 1.00 (ref) |  |
| Yes | 1852 | 0.66 (0.57, 0.77) | <0.001 | 1770 | 0.84 (0.72, 0.98) | 0.009 |
| **Hypertension** |  |  |  |  |  |  |
| No | 672 | 1.00 (ref) |  | 657 | 1.00 (ref) |  |
| Yes | 3188 | 0.83 (0.69, 1.01) | 0.06 | 2987 | 1.09 (0.90, 1.33) | 0.38 |
| **Hypercolesterolaemia** |  |  |  |  |  |  |
| No | 1071 | 1.00 (ref) |  | 1018 | 1.00 (ref) |  |
| Yes | 2789 | 1.02 (0.87, 1.20) | 0.74 | 2626 | 1.19 (1.01, 1.40) | 0.04 |
| **Family history of premature CHD** | |  |  |  |  |  |
| No | 2994 | 1.00 (ref) |  | 2836 | 1.00 (ref) |  |
| Yes | 866 | 0.82 (0.70, 0.97) | 0.02 | 808 | 0.83 (0.69, 0.98) | 0.03 |
| **Depression** |  |  |  |  |  |  |
| No | 3183 | 1.00 (ref) |  | 3010 | 1.00 (ref) |  |
| Yes | 677 | 1.06 (0.88, 1.27) | 0.54 | 634 | 0.96 (0.79, 1.16) | 0.66 |
| **Obesity** |  |  |  |  |  |  |
| No | 2101 | 1.00 (ref) |  | 1956 | 1.00 (ref) |  |
| Yes | 1759 | 1.05 (0.89, 1.25) | 0.55 | 1688 | 1.02 (0.86, 1.22) | 0.80 |
| **SBP** (per 5 mmHg) | 3860 | 1.02 (1.00, 1.04) | 0.09 | 3644 | 1.00 (0.98, 1.02) | 0.91 |
| **DBP** (per 5 mmHg) | 3860 | 0.99 (0.96, 1.03) | 0.79 | 3644 | 1.00 (0.96, 1.04) | 0.94 |
| **Waist circumference** (per 5 cm) | 3860 | 0.94 (0.90, 0.97) | <0.001 | 3644 | 0.92 (0.88, 0.95 | <0.001 |
| **Physical activity** (MET-min/d) ^c,e^ | |  |  |  |  |  |
| T1 (low) | 1208 | 1.00 (ref) |  | 1152 | 1.00 (ref) |  |
| T2 | 1309 | 1.07 (0.91, 1.26) |  | 1229 | 1.24 (1.05, 1.48) |  |
| T3 (high) | 1343 | 1.37 (1.15, 1.63) | <0.001 | 1263 | 1.29 (1.08, 1.54) | <0.001 |
| **Smoking Status** |  |  |  |  |  |  |
| Never | 2392 | 1.00 (ref) |  | 2264 | 1.00 (ref) |  |
| Former | 504 | 0.94 (0.75, 1.19) | 0.62 | 471 | 0.89 (0.70, 1.14) | 0.37 |
| Current | 964 | 0.95 (0.78, 1.15) | 0.58 | 909 | 1.07 (0.87, 1.31) | 0.52 |
| **Total energy intake** (kcal/day) ^c,f^ |  |  |  |  |  |  |
| Q1 (low) | 946 | 1.00 (ref) |  | 872 | 1.00 (ref) |  |
| Q2 | 971 | 1.09 (0.90, 1.32) |  | 910 | 1.29 (1.06, 1.57) |  |
| Q3 | 980 | 1.24 (1.03, 1.51) |  | 920 | 1.39 (1.14, 1.69) |  |
| Q4 (high) | 963 | 1.07 (0.88, 1.38) | 0.41 | 942 | 1.33 (1.09, 1.63) | 0.008 |
| **Alcohol other than wine** (g/day) |  |  |  |  |  |  |
| <10 men, <5 women | 2242 | 1.00 (ref) |  | 2118 | 1.00 (ref) |  |
| 10-50 men, 5-10 women | 560 | 0.85 (0.69, 1.04) | 0.13 | 525 | 0.88 (0.71, 1.09) | 0.25 |
| >50 men, >10 women | 1058 | 0.90 (0.77, 1.06) | 0.22 | 1001 | 1.03 (0.87, 1.21) | 0.77 |
| **14-point adherence score** ^a^ | | |  |  |  |  |
| <11 | 3159 | 1.00 (ref) |  | 2950 | 1.00 (ref) |  |
| ≥11 | 701 | 2.34 (1.94, 2.83) | <0.001 | 694 | 1.96 (1.62, 2.37) | <0.001 |
| **Study Design Features** |  |  |  |  |  |  |
| **Intervention Group** |  |  |  |  |  |  |
| MedDiet+Nuts | 1776 | 1.00 (ref) |  | 1631 | 1.00 (ref) |  |
| MedDiet+EVOO | 2084 | 0.78 (0.69, 0.90) | <0.001 | 2013 | 0.77 (0.67, 0.80) | <0.001 |
| **Total workload of center (person-years)** ^e,g^ | | |  |  |  |  |
| Q1 (low) | 1081 | 1.00 (ref) |  | 1071 | 1.00 (ref) |  |
| Q2 | 1026 | 1.56 (1.30, 1.88) |  | 915 | 0.56 (0.47, 0.68) |  |
| Q3 | 1130 | 1.85 (1.53, 2.23) |  | 1067 | 0.71 (0.59, 0.85) |  |
| Q4 (high) | 623 | 1.30 (1.05, 1.60) | <0.001 | 591 | 1.84 (1.44, 2.34) | 0.003 |
| ^a^ ORs < 1 imply poorer adherence. ORs > 1 imply better adherence. A validated MedDiet adherence assessment tool was used. 1 point was added for each item in adherence with the traditional MedDiet. High adherence = adherence with ≥11 items on 14-point dietary adherence score. Low adherence = adherence with <11 items. ^b^ All models are from logistic regression analysis. Multivariate models are mutually adjusted for all characteristics displayed in this table including total CVD risk score but excluding individual CVD risk factors (type 2 diabetes, hypertension, hypercholesterolaemia, family history of pre-mature CHD, depression, obesity). When an individual CVD risk factors was the exposure of interest, the model was mutually adjusted for other individual CVD risk factors but not total CVD risk score.^c^ P-values for trend were calculated by assigning the median value to each category and treating the resulting variable as continuous. ^d^ Total CVD risk score calculated by summing the following CVD risk factors: type 2 diabetes, hypertension, high blood cholesterol, family history of premature CHD, depression, obesity  ^e^ Tertiles of physical activity (MET-min/d): T1: <108; T2: 108-268; T3: ≥268. ^f^ Quartiles of energy intake (kcal/d), by sex: Men: Q1: <2051; Q2: 2051-<2394; Q3:2934-<2801; Q4: ≥2801. Women: Q1: <1786; Q2: 1786-<2109; Q3: 2109-<2465; Q4: ≥2465. ^g^ Measured in quartiles of person years at center. After 2 years: Q1: 270-<633. Q2: 633-<1069. Q3: 1069-<1273. Q4: ≥1273. After 3 years: Q1: 414-<906. Q2: 906-<1597. Q3: 1597-<1820. Q4: ≥1820. | | | | | | |

**Additional file 1: Table S2. Odds of high adherence with the MedDiet intervention using alternate adherence score cut-points ^a^**.

|  | | **Multivariate OR (95% CI) for dietary adherence ^b^** | | | | | | | | | | |
| --- | --- | --- | --- | --- | --- | --- | --- | --- | --- | --- | --- | --- |
|  | | **1 Year** | | | | | **4 Years** | | | | | |
| **Demographic Characteristics** | | n | <10 vs. ≥10 points | *p* | <12 vs. ≥12 points | *p* | | n | <10 vs. ≥10 points | *p* | <12 vs. ≥12 points | *p* |
| **Sex** | |  |  |  |  |  | |  |  |  |  |  |
| Men | | 1820 | 1.00 (ref) |  | 1.00 (ref) |  | | 1103 | 1.00 (ref) |  | 1.00 (ref) |  |
| Women | | 3278 | 0.73 (0.58, 0.92) | 0.007 | 0.77 (0.62, 0.96) | 0.02 | | 1350 | 0.88 (0.63, 1.240 | 0.46 | 0.81 (0.60, 1.10) | 0.18 |
| **Age at baseline** (years) | |  |  |  |  |  | |  |  |  |  |  |
| <65 | | 1627 | 1.00 (ref) |  | 1.00 (ref) |  | | 868 | 1.00 (ref) |  | 1.00 (ref) |  |
| ≥65 | | 2571 | 0.86 (0.72, 1.02) | 0.09 | 0.84 (0.71, 1.00) | 0.05 | | 1485 | 0.97 (0.75, 1.25) | 0.81 | 0.81 (0.65, 1.01) | 0.06 |
| **Educational level** | |  |  |  |  |  | |  |  |  |  |  |
| University or higher | | 339 | 1.00 (ref) |  | 1.00 (ref) |  | | 173 | 1.00 (ref) |  | 1.00 (ref) |  |
| Secondary school | | 643 | 1.01 (0.73, 1.40) | 0.93 | 0.88 (0.65, 1.17) | 0.37 | | 356 | 0.58 (0.34, 0.97) | 0.04 | 1.07 (0.73, 1.59) | 0.72 |
| Primary School | | 3109 | 0.87 (0.65, 1.16) | 0.33 | 0.88 (0.68, 1.14) | 0.34 | | 1768 | 0.65 (0.40, 1.05) | 0.08 | 1.01 (0.71, 1.43) | 0.97 |
| Less than primary school | | 107 | 0.72 (0.44, 1.20) | 0.21 | 0.83 (0.49, 1.42) | 0.50 | | 57 | 0.70 (0.32, 1.50) | 0.35 | 0.84 (0.40, 1.75) | 0.64 |
| **Occupation** | |  |  |  |  |  | |  |  |  |  |  |
| Retired | | 2234 | 1.00 (ref) |  | 1.00 (ref) |  | | 1127 | 1.00 (ref) |  | 1.00 (ref) |  |
| Working | | 509 | 0.70 (0.54, 0.91) | 0.007 | 0.76 (0.59, 0.97) | 0.03 | | 258 | 0.96 (0.65, 1.42) | 0.83 | 0.93 (0.67, 1.28) | 0.64 |
| Housewife | | 1324 | 1.04 (0.86, 1.27) | 0.69 | 1.21 (0.99, 1.48) | 0.06 | | 802 | 0.93 (0.70, 1.24) | 0.62 | 0.96 (0.74, 1.25) | 0.75 |
| Unemployed/unable to work | | 131 | 0.78 (0.51, 1.18) | 0.24 | 0.73 (0.47, 1.13) | 0.16 | | 66 | 0.71 (0.39, 1.30) | 0.27 | 0.75 (0.42, 1.35) | 0.34 |
| **Marital Status** | |  |  |  |  |  | |  |  |  |  |  |
| Married | | 3266 | 1.00 (ref) |  | 1.00 (ref) |  | | 1824 | 1.00 (ref) |  | 1.00 (ref) |  |
| Single | | 157 | 1.06 (0.72, 1.55) | 0.78 | 1.08 (0.75, 1.55) | 0.69 | | 88 | 0.84 (0.49, 1.46) | 0.55 | 0.82 (0.51, 1.34) | 0.43 |
| Widowed | | 668 | 0.96 (0.78, 1.18) | 0.67 | 0.87 (0.70, 1.08) | 0.21 | | 399 | 0.85 (0.64, 1.13) | 0.26 | 0.81 (0.62, 1.06) | 0.13 |
| Divorced or separated | | 107 | 0.86 (0.55, 1.32) | 0.48 | 1.00 (0.63, 1.60) | 0.99 | | 42 | 1.73 (0.76, 3.92) | 0.19 | 1.03 (0.51, 2.09) | 0.93 |
| **Number of people in household** | | 4198 | 1.01 (0.95, 1.07) | 0.80 | 1.00 (0.95, 1.06) | 0.92 | | 2353 | 1.03 (0.94, 1.13) | 0.50 | 1.00 (0.93, 1.07) | 0.95 |
| **Health-Related Characteristics at Baseline** | | | |  |  |  | |  |  |  |  |  |
| **Number of CVD Risk Factors** ^c,d^ | | |  |  |  |  | |  |  |  |  |  |
| 0-1 | | 285 | 1.00 (ref) |  | 1.00 (ref) |  | | 204 | 1.00 (ref) |  | 1.00 (ref) |  |
| 2 | | 1237 | 0.98 (0.71, 1.35) |  | 0.97 (0.73, 1.28) |  | | 734 | 0.79 (0.51, 1.23) |  | 1.04 (0.74, 1.46) |  |
| 3 | | 1632 | 1.01 (0.73, 1.40) |  | 0.85 (0.64, 1.13) |  | | 884 | 0.93 (0.59, 1.44) |  | 1.04 (0.74, 1.47) |  |
| 4 | | 824 | 0.88 (0.62, 1.24) |  | 0.68 (0.50, 0.94) |  | | 429 | 0.81 (0.0, 1.30) |  | 0.75 (0.51, 1.11) |  |
| 5-6 | | 220 | 0.80 (0.52, 1.23) | 0.18 | 0.71 (0.46, 1.09) | 0.003 | | 102 | 0.67 (0.36, 1.25) | 0.41 | 0.66 (0.37, 1.19) | 0.05 |
| **Type 2 Diabetes** | |  |  |  |  |  | |  |  |  |  |  |
| No | | 2199 | 1.00 (ref) |  | 1.00 (ref) |  | | 1221 | 1.00 (ref) |  | 1.00 (ref) |  |
| Yes | | 1999 | 0.78 (0.66, 0.91) | 0.002 | 0.76 (0.65, 0.89) | 0.001 | | 1132 | 0.86 (0.69, 1.08) | 0.20 | 0.76 (0.62, 0.93) | 0.008 |
| **Hypertension** | |  |  |  |  |  | |  |  |  |  |  |
| No | | 757 | 1.00 (ref) |  | 1.00 (ref) |  | | 447 | 1.00 (ref) |  | 1.00 (ref) |  |
| Yes | | 3441 | 0.97 (0.80, 1.19) | 0.80 | 0.83 (0.68, 1.00) | 0.06 | | 1906 | 0.89 (0.67, 1.19) | 0.43 | 1.04 (0.81, 1.34) | 0.77 |
| **Hypercolesterolaemia** | |  |  |  |  |  | |  |  |  |  |  |
| No | | 1169 | 1.00 (ref) |  | 1.00 (ref) |  | | 748 | 1.00 (ref) |  | 1.00 (ref) |  |
| Yes | | 3029 | 1.03 (0.87, 1.22) | 0.71 | 1.04 (0.88, 1.22) | 0.68 | | 1605 | 1.35 (1.07, 1.69) | 0.01 | 1.01 (0.82, 1.24) | 0.95 |
| **Family history of premature CHD** | | |  |  |  |  | |  |  |  |  |  |
| No | | 3237 | 1.00 (ref) |  | 1.00 (ref) |  | | 1871 | 1.00 (ref) |  | 1.00 (ref) |  |
| Yes | | 961 | 0.84 (0.71, 1.00) | 0.05 | 0.75 (0.63, 0.89) | 0.001 | | 482 | 0.70 (0.54, 0.89) | 0.005 | 0.75 (0.59, 0.95) | 0.02 |
| **Depression** | |  |  |  |  |  | |  |  |  |  |  |
| No | | 3481 | 1.00 (ref) |  | 1.00 (ref) |  | | 1975 | 1.00 (ref) |  | 1.00 (ref) |  |
| Yes | | 717 | 0.94 (0.77, 1.14) | 0.53 | 0.87 (0.71, 1.06) | 0.17 | | 378 | 0.78 (0.59, 1.03) | 0.08 | 0.78 (0.60, 1.01) | 0.06 |
| **Obesity** | |  |  |  |  |  | |  |  |  |  |  |
| No | | 2282 | 1.00 (ref) |  | 1.00 (ref) |  | | 1302 | 1.00 (ref) |  | 1.00 (ref) |  |
| Yes | | 1916 | 1.07 (0.89, 1.28) | 0.47 | 0.98 (0.82, 1.16) | 0.80 | | 1051 | 1.15 (0.89, 1.49) | 0.29 | 0.99 (0.79, 1.25) | 0.93 |
| **SBP** (per 5 mmHg) | | 4198 | 1.01 (0.99, 1.03) | 0.31 | 1.03 (1.01, 1.04) | 0.01 | | 2353 | 0.98 (0.96, 1.01) | 0.31 | 1.01 (0.99, 1.04) | 0.40 |
| **DBP** (per 5 mmHg) | | 4198 | 1.00 (0.96, 1.04) | 0.93 | 1.00 (0.96, 1.04) | 0.94 | | 2353 | 1.00 (0.95, 1.06) | 0.96 | 1.01 (0.96, 1.06) | 0.73 |
| **Waist circumference** (per 5 cm) | | 4198 | 0.92 (0.89, 0.96) | <0.001 | 0.92 (0.88, 0.95) | <0.001 | | 2353 | 0.93 (0.88, 0.98) | 0.01 | 0.95 (0.90, 1.00) | 0.05 |
| **Physical activity** (MET-min/d) ^c,e^ | | |  |  |  |  | |  |  |  |  |  |
| T1 (low) | 1332 | | 1.00 (ref) |  | 1.00 (ref) |  | | 703 | 1.00 (ref) |  | 1.00 (ref) |  |
| T2 | 1414 | | 1.17 (0.98, 1.39) |  | 1.21 (1.01, 1.44) |  | | 785 | 1.18 (0.93, 1.51) |  | 1.42 (1.12, 1.79) |  |
| T3 (high) | 1452 | | 1.42 (1.18, 1.72) | <0.001 | 1.52 (1.26, 1.82) | <0.001 | | 865 | 1.75 (1.33, 2.29) | <0.001 | 1.69 (1.33, 2.15) | <0.001 |
| **Smoking Status** |  | |  |  |  |  | |  |  |  |  |  |
| Never | 2576 | | 1.00 (ref) |  | 1.00 (ref) |  | | 1482 | 1.00 (ref) |  | 1.00 (ref) |  |
| Former | 574 | | 0.70 (0.55, 0.89) | 0.004 | 0.82 (0.65, 1.04) | 0.11 | | 320 | 1.05 (0.72, 1.52) | 0.81 | 0.85 (0.62, 1.15) | 0.29 |
| Current | 1048 | | 0.93 (0.75, 1.15) | 0.48 | 1.13 (0.93, 1.38) | 0.22 | | 551 | 0.99 (0.73, 1.34) | 0.94 | 0.82 (0.63, 1.06) | 0.13 |
| **Total energy intake** (kcal/day) ^c,f^ | | |  |  |  |  | |  |  |  |  |  |
| Q1 (low) | 1017 | | 1.00 (ref) |  | 1.00 (ref) |  | | 517 | 1.00 (ref) |  | 1.00 (ref) |  |
| Q2 | 1038 | | 1.24 (1.01, 1.52) |  | 1.20 (0.98, 1.47) |  | | 551 | 1.04 (0.78, 1.39) |  | 1.20 (0.92, 1.57) |  |
| Q3 | 1078 | | 1.20 (0.98, 1.47) |  | 1.30 (1.06, 1.58) |  | | 596 | 1.46 (1.08, 1.97) |  | 1.27 (0.97, 1.66) |  |
| Q4 (high) | 1065 | | 1.10 (0.90, 1.35) | 0.50 | 1.36 (1.11, 1.67) | <0.003 | | 689 | 1.17 (0.88, 1.57) | 0.15 | 1.16 (0.89, 1.52) | 0.36 |
| **Alcohol other than wine** (g/day) | | |  |  |  |  | |  |  |  |  |  |
| <10 men, <5 women | 2429 | | 1.00 (ref) |  | 1.00 (ref) |  | | 1360 | 1.00 (ref) |  | 1.00 (ref) |  |
| 10-50 men, 5-10 women | 606 | | 0.77 (0.61, 0.96) | 0.02 | 0.96 (0.78, 1.18) | 0.70 | | 344 | 0.83 (0.60, 1.14) | 0.25 | 0.89 (0.68, 1.18) | 0.43 |
| >50 men, >10 women | 1163 | | 0.94 (0.79, 1.12) | 0.51 | 0.95 (0.80, 1.12) | 0.52 | | 649 | 1.20 (0.93, 1.55) | 0.16 | 1.16 (0.93, 1.45) | 0.18 |
| **14-point adherence score** ^a^ | | | |  |  |  | |  |  |  |  |  |
| <11 | 3416 | | 1.00 (ref) |  | 1.00 (ref) |  | | 1864 | 1.00 (ref) |  | 1.00 (ref) |  |
| ≥11 | 782 | | 4.27 (3.28, 5.57) | <0.001 | 2.57 (2.18, 3.04) | <0.001 | | 489 | 2.53 (1.85, 3.48) | <0.001 | 1.71 (1.38, 2.13) | <0.001 |
| **Study Design Features** |  | |  |  |  |  | |  |  |  |  |  |
| **Intervention Group** |  | |  |  |  |  | |  |  |  |  |  |
| MedDiet+Nuts | 1962 | | 1.00 (ref) |  | 1.00 (ref) |  | | 1027 | 1.00 (ref) |  | 1.00 (ref) |  |
| MedDiet+EVOO | 2,236 | | 0.72 (0.63, 0.84) | <0.001 | 0.74 (0.65, 0.85) | <0.001 | | 1326 | 0.75 (0.61, 0.93) | 0.007 | 0.80 (0.67, 0.96) | 0.02 |
| **Total workload of center (person-years)** ^e,g^ | | | |  |  |  | |  |  |  |  |  |
| Q1 (low) | 1247 | | 1.00 (ref) |  | 1.00 (ref) |  | | 624 | 1.00 (ref) |  | 1.00 (ref) |  |
| Q2 | 1133 | | 0.60 (0.48, 0.74) |  | 0.44 (0.35, 0.55) |  | | 361 | 0.63 (0.48, 0.83) |  | 0.81 (0.62, 1.05) |  |
| Q3 | 1168 | | 0.70 (0.57, 0.87) |  | 0.66 (0.53, 0.81) |  | | 804 | 0.85 (0.66, 1.11) |  | 0.84 (0.66, 1.07) |  |
| Q4 (high) | 650 | | 3.06 (2.15, 4.35) | <0.001 | 2.24 (1.77, 2.83) | <0.001 | | 564 | 3.04 (2.08, 4.43) | <0.001 | 2.15 (1.65, 2.79) | <0.001 |
| ^a^ Control group was excluded. ORs < 1 imply poorer adherence. ORs > 1 imply better adherence. A validated MedDiet adherence assessment tool was used. 1 point was added for each item in adherence with the traditional MedDiet. High adherence = adherence with ≥10 or ≥12 items on 14-point dietary adherence score. Low adherence = adherence with <10 or <12 items.  ^b^ All models are from logistic regression analysis. Multivariate models are mutually adjusted for all characteristics displayed in this table including total CVD risk score but excluding individual CVD risk factors (type 2 diabetes, hypertension, hypercholesterolaemia, family history of pre-mature CHD, depression, obesity). When an individual CVD risk factors was the exposure of interest, the model was mutually adjusted for other individual CVD risk factors but not total CVD risk score. ^c^ P-values for trend were calculated by assigning the median value to each category and treating the resulting variable as continuous. ^d^ Risk score calculated by summing the following CVD risk factors: type 2 diabetes, hypertension, high blood cholesterol, family history of premature CHD, depression, obesity. ^e^ Tertiles of physical activity (MET-min/d): T1: <108; T2: 108-268; T3: ≥268. ^f^ Quartiles of energy intake (kcal/d), by sex: Men: Q1: <2051; Q2: 2051-<2394; Q3:2934-<2801; Q4: ≥2801. Women: Q1: <1786; Q2: 1786-<2109; Q3: 2109-<2465; Q4: ≥2465. ^g^ Measured in quartiles of person years at center. After 1 Year: Q1: 133-<352; Q2: 352-<537; Q3: 537-<650; Q4: ≥650. After 4 years: Q1: 893-<1220; Q2: 1220-<2175; Q3: 2175-<2384; Q4: ≥2384. | | | | | | | | | | | | |

**Additional file 1: Table S3. Odds of adherence with olive oil and nut consumption after 1 and 4 years of follow-up^a^**.

|  | **1 Year ^b^** | | **4 Years ^b^** | |
| --- | --- | --- | --- | --- |
| **Intervention Group** | ≥4 Tbsp Olive Oil/d^c^ | p-value | ≥4 Tbsp Olive Oil/d^c^ | p-value |
| **Nuts** | 1.00 (ref) |  | 1.00 (ref) |  |
| **Olive Oil** | 4.72 (3.65, 6.12) | <0.001 | 5.66 (4.07, 7.86) | <0.001 |
|  | Olive Oil as Main Culinary Fat^d^ |  | Olive Oil as Main Culinary Fat^d^ |  |
| **Nuts** | 1.00 (ref) |  | 1.00 (ref) |  |
| **Olive Oil** | 5.06 (2.73, 9.35) | <0.001 | 9.57 (3.57, 25.67) | <0.001 |
|  | ≥3 Servings Nuts/wk^e^ |  | ≥3 Servings Nuts/wk^e^ |  |
| **Olive Oil** | 1.00 (ref) |  | 1.00 (ref) |  |
| **Nuts** | 21.89 (17.63, 27.19) | <0.001 | 19.26 (14.97, 24.77) | <0.001 |
| ^a^ Control group was excluded. ORs < 1 imply poorer adherence. ORs > 1 imply better adherence. A validated MedDiet adherence assessment tool was used. 1 point was added for each item in adherence with the traditional MedDiet.  ^b^ All models are from logistic regression analysis. Multivariate models are adjusted for the following characteristics at baseline: sex (men, women), age at baseline (<65 years, ≥65 years), highest educational level attained (university or higher, secondary school, primary school, less than primary school), occupation (retired, working, housewife, unemployed/unable to work), marital status (married, single, widowed, divorced or separated), number of people in household, number of baseline CVD risk factors (0-1, 2, 3, 4, 5-6), type 2 diabetes (yes, no) hypertension (yes, no), hypercholesterolemia (yes, no), family history of premature CHD (yes, no), depression (yes, no), obesity (yes, no), systolic blood pressure (yes, no), diastolic blood pressure (yes, no), physical activity (MET-min/day, tertiles), smoking status (never, former, current), total energy intake (quartiles), alcohol other than wine (grams per day, low: <10 men, <5 women, medium: 10-50 men, 5-10 women, high: >50 men, >10 women), annual work load (person years) per center (quartiles).  ^c^ Models are additionally adjusted for adherence to ≥3 servings of nuts per week at same visit and adherence to ≥4 tablespoons of olive oil per day at baseline.  ^d^ Models are additionally adjusted for adherence to ≥3 servings of nuts per week at same visit and adherence to olive oil as main culinary fat at baseline.  ^e^ Models are additionally adjusted for adherence to ≥4 tablespoons of olive oil per day and olive oil as main culinary fat at same visit, and adherence to ≥3 servings of nuts per week at baseline. | | | | |

**Additional file 1: Table S4. Odds of high adherence with the MedDiet intervention at one year^a^**, restricting the analyses to those participants recruited before 2006.

|  | **Multivariate OR (95% CI) for dietary adherence**  **(≥11 vs. <11 points)^b^** | | |
| --- | --- | --- | --- |
| **Characteristics at baseline** | n |  | *p* |
| **Sex** |  |  |  |
| Men | 1274 | 1.00 (ref) |  |
| Women | 1656 | 0.89 (0.63, 1.27) | 0.53 |
| **Age at baseline** (years) |  |  |  |
| <65 | 1071 | 1.00 (ref) |  |
| ≥65 | 1859 | 1.01 (0.76, 1.34) | 0.94 |
| **Educational level** |  |  |  |
| University or higher | 227 | 1.00 (ref) |  |
| Secondary school | 435 | 1.06 (0.66, 1.73) | 0.66 |
| Primary School | 2196 | 0.97 (0.63, 1.49) | 0.63 |
| Less than primary school | 72 | 0.81 (0.35, 1.86) | 0.35 |
| **Occupation** |  |  |  |
| Retired | 1539 | 1.00 (ref) |  |
| Working | 317 | 0.77 (0.52, 1.13) | 0.50 |
| Housewife | 996 | 1.12 (0.81, 1.57) | 0.13 |
| Unemployed/unable to work | 78 | 1.03 (0.56, 1.90) | 0.78 |
| **Marital Status** |  |  |  |
| Married | 2279 | 1.00 (ref) |  |
| Single | 108 | 0.81 (0.44, 1.50) | 0.50 |
| Widowed | 485 | 0.75 (0.53, 1.08) | 0.13 |
| Divorced or separated | 58 | 1.09 (0.59, 2.01) | 0.78 |
| **Number of people in household** | 2930 | 0.98 (0.89, 1.08) | 0.68 |
| **Number of CVD Risk Factors** ^c,d^ |  |  |  |
| 0-1 | 242 | 1.00 (ref) |  |
| 2 | 893 | 1.20 (0.61, 2.35) |  |
| 3 | 1131 | 1.35 (0.69, 2.66) |  |
| 4 | 529 | 1.46 (0.72, 2.94) |  |
| 5-6 | 135 | 1.01 (0.45, 2.28) | 0.68 |
| **Type 2 Diabetes** |  |  |  |
| No | 1480 | 1.00 (ref) |  |
| Yes | 1450 | 0.70 (0.54, 0.92) | 0.01 |
| **Hypertension** |  |  |  |
| No | 569 | 1.00 (ref) |  |
| Yes | 2361 | 1.25 (0.88, 1.78) | 0.21 |
| **Hypercolesterolaemia** |  |  |  |
| No | 924 | 1.00 (ref) |  |
| Yes | 2006 | 1.22 (0.89, 1.67) | 0.22 |
| **Family history of premature CHD** |  |  |  |
| No | 2320 | 1.00 (ref) |  |
| Yes | 610 | 1.09 (0.83, 1.44) | 0.53 |
| **Depression** |  |  |  |
| No | 2474 | 1.00 (ref) |  |
| Yes | 456 | 1.10 (0.81, 1.48) | 0.55 |
| **Obesity** |  |  |  |
| No | 1594 | 1.00 (ref) |  |
| Yes | 1336 | 1.00 (0.74, 1.34) | 0.98 |
| **SBP** (per 5 mmHg) | 2930 | 1.01 (0.97, 1.04) | 0.69 |
| **DBP** (per 5 mmHg) | 2930 | 0.97 (0.91, 1.03) | 0.34 |
| **Waist circumference** (per 5 cm) | 2930 | 0.92 (0.86, 0.98) | 0.008 |
| **Physical activity** (MET-min/d) ^c,e^ |  |  |  |
| T1 (low) | 1803 | 1.00 (ref) |  |
| T2 | 412 | 1.14 (0.86, 1.52) |  |
| T3 (high) | 715 | 1.25 (0.93, 1.69) | 0.15 |
| **Smoking Status** |  |  |  |
| Never | 1803 | 1.00 (ref) |  |
| Former | 412 | 0.80 (0.54, 1.18) | 0.26 |
| Current | 715 | 1.04 (0.75, 1.44) | 0.81 |
| **Total energy intake** (kcal/day) ^c, f^ |  |  |  |
| Q1 (low) | 676 | 1.00 (ref) |  |
| Q2 | 697 | 1.37 (1.00, 1.88) |  |
| Q3 | 752 | 1.16 (0.83, 1.61) |  |
| Q4 (high) | 805 | 1.31 (0.92, 1.85) | 0.24 |
| **Alcohol other than wine** (g/day) |  |  |  |
| <10 men, <5 women | 1702 | 1.00 (ref) |  |
| 10-50 men, 5-10 women | 438 | 0.91 (0.63, 1.33) | 0.64 |
| >50 men, >10 women | 790 | 0.89 (0.68, 1.18) | 0.43 |
| **14-point adherence score** ^a^ | | |  |
| <11 | 2359 | 1.00 (ref) |  |
| ≥11 | 571 | 2.31 (1.68, 3.19) | <0.001 |
| **Study Design Features** |  |  |  |
| **Intervention Group** |  |  |  |
| MedDiet+Nuts | 1635 | 1.00 (ref) |  |
| MedDiet+EVOO | 1295 | 0.67 (0.53, 0.85) | <0.001 |
| **Total workload of center (person-years)** ^c, g^ | |  |  |
| Q1 (low) | 717 | 1.00 (ref) |  |
| Q2 | 722 | 1.27 (0.96, 1.69) |  |
| Q3 | 1076 | 1.09 (0.67, 1.76) |  |
| Q4 (high) | 415 | 1.61 (1.16, 2.25) | 0.009 |
| ^a^ ORs < 1 imply poorer adherence. ORs > 1 imply better adherence. A validated MedDiet adherence assessment tool was used. 1 point was added for each item in adherence with the traditional MedDiet. High adherence = adherence with ≥11 items on 14-point dietary adherence score. Low adherence = adherence with <11 items. ^b^ All models are from logistic regression analysis. Multivariate models are mutually adjusted for all characteristics displayed in this table including total CVD risk score but excluding individual CVD risk factors (type 2 diabetes, hypertension, hypercholesterolaemia, family history of pre-mature CHD, depression, obesity). When an individual CVD risk factors was the exposure of interest, the model was mutually adjusted for other individual CVD risk factors but not total CVD risk score. ^c^ P-values for trend were calculated by assigning the median value to each category and treating the resulting variable as continuous. ^d^ CVD risk score was calculated by summing the following CVD risk factors: type 2 diabetes, hypertension, high blood cholesterol, family history of premature CHD, depression, obesity. ^e^ Tertiles of physical activity (MET-min/d): T1: <108; T2: 108-268; T3: ≥268. ^f^ Quartiles of energy intake (kcal/d), by sex: Men: Q1: <2051; Q2: 2051-<2394; Q3:2934-<2801; Q4: ≥2801. Women: Q1: <1786; Q2: 1786-<2109; Q3: 2109-<2465; Q4: ≥2465. ^g^ Measured in quartiles of person years at center. After 1 Year: Q1: 133-<352; Q2: 352-<537; Q3: 537-<650; Q4: ≥650. | | | |

**Additional file 1: Table S5. Adherence at one year of follow-up according to a 14-point dietary adherence score and year of recruitment into PREDIMED^a^**

|  | **Recruited before November, 2006** | |  |  | | **Recruited after**  **November, 2006** | |
| --- | --- | --- | --- | --- | --- | --- | --- |
| Adherence**^b^** | Low  (n=1243) | High (n=1687) |  |  | | Low (n=682) | High  (n=586) |
| **Demographic Characteristics** | % or mean (SD) | |  |  | | % or mean (SD) | |
| **Women** | 60.2 | 53.8 |  |  | | 57.2 | 56.7 |
| **Age at baseline** (years) | 67.3 (6.2) | 67.1 (6.0) |  |  | | 66.2 (5.9) | 66.3 (5.6) |
| **Educational level** |  |  |  |  | |  |  |
| University | 6.7 | 8.7 |  |  | | 8.8 | 9.3 |
| Secondary school | 13.9 | 15.9 |  |  | | 16.4 | 17.2 |
| Primary school | 76.5 | 73.3 |  |  | | 71.5 | 71.3 |
| Less than primary school | 2.9 | 2.2 |  |  | | 3.3 | 2.3 |
| **Occupation** |  |  |  |  | |  |  |
| Retired | 49.6 | 54.7 |  |  | | 55.1 | 54.4 |
| Working | 11.3 | 10.5 |  |  | | 16.6 | 13.5 |
| Housewife | 35.5 | 32.9 |  |  | | 24.2 | 27.8 |
| Unemployed/unable to work | 3.6 | 2.0 |  |  | | 4.1 | 4.3 |
| **Marital Status** |  |  |  |  | |  |  |
| Married | 76.8 | 78.5 |  |  | | 76.1 | 79.9 |
| Single | 3.5 | 3.8 |  |  | | 4.1 | 3.6 |
| Widowed | 17.3 | 16.0 |  |  | | 16.0 | 12.6 |
| Divorced or separated | 2.4 | 1.7 |  |  | | 3.8 | 3.9 |
| **Health-Related Characteristics at Baseline** | |  |  |  | |  |  |
| **Number of people in household** | 1.7 (1.3) | 1.7 (1.5) |  |  | | 1.6 (1.4) | 1.6 (1.1) |
| **Number of CVD Risk Factors^c^** |  |  |  |  | |  |  |
| 0-1 | 7.0 | 9.2 |  |  | | 3.7 | 3.1 |
| 2 | 27.3 | 32.8 |  |  | | 26.8 | 27.5 |
| 3 | 39.3 | 38.1 |  |  | | 38.6 | 40.6 |
| 4 | 21.3 | 15.7 |  |  | | 23.0 | 23.6 |
| 5-6 | 5.1 | 4.3 |  |  | | 7.9 | 5.3 |
| **Type 2 Diabetes** | 53.5 | 46.5 |  |  | | 48.0 | 36.9 |
| **Hypertension** | 81.6 | 79.9 |  |  | | 83.4 | 87.2 |
| **Hypercholesterolemia** | 66.8 | 69.7 |  |  | | 78.0 | 83.8 |
| **Family history of premature CHD** | 80.6 | 19.4 |  |  | | 26.4 | 29.2 |
| **Depression** | 16.8 | 14.6 |  |  | | 20.5 | 20.7 |
| **Obesity** | 49.2 | 43.0 |  |  | | 48.7 | 42.3 |
| **SBP** (mmHg) | 149.3 (20.8) | 150.7 (20.9) |  |  | | 147.1 (20.5) | 146.9 (19.4) |
| **DBP** (mmHg) | 83.1 (10.7) | 84.1 (11.3) |  |  | | 82.7 (10.9) | 81.9 (10.3) |
| **Waist circumference** (cm) | 101.3 (10.0) | 99.1 (10.7) |  |  | | 101.2 (10.1) | 99.2 (10.5) |
| **Physical activity** (MET-min/d)**^d^** |  |  |  |  | |  |  |
| T1 (low) | 37.3 | 27.2 |  |  | | 33.9 | 29.2 |
| T2 | 34.0 | 32.9 |  |  | | 34.9 | 35.7 |
| T3 (high) | 28.6 | 29.9 |  |  | | 31.2 | 35.2 |
| **Smoking Status** |  |  |  |  | |  |  |
| Never | 62.7 | 60.7 |  |  | | 61.1 | 60.8 |
| Former | 15.0 | 13.3 |  |  | | 13.9 | 11.4 |
| Current | 22.3 | 26.0 |  |  | | 24.9 | 27.8 |
| **Total energy intake** (kcal/day)^e^ | |  |  |  | |  |  |
| Q1 (low) | 26.2 | 20.5 |  |  | | 29.2 | 23.9 |
| Q2 | 24.0 | 23.7 |  |  | | 25.2 | 29.0 |
| Q3 | 24.9 | 26.6 |  |  | | 26.0 | 26.1 |
| Q4 (high) | 24.8 | 29.2 |  |  | | 19.7 | 21.0 |
| **Alcohol other than wine** (g/day) | | | | |  |  |  |
| <10 men, <5 women | 58.2 | 57.7 |  |  | | 57.3 | 57.3 |
| 10-50 men, 5-10 women | 14.7 | 15.2 |  |  | | 13.3 | 13.1 |
| >50 men, > 10 women | 27.1 | 27.0 |  |  | | 29.3 | 29.5 |
| **14-point adherence score^b^** | 8.2 (1.8) | 9.4 (1.9) |  |  | | 8.2 (1.9) | 9.2 (1.8) |
| **Intervention Design Features** |  |  |  |  | |  |  |
| **Intervention Group** |  |  |  |  | |  |  |
| MedDiet+EVOO | 61.6 | 51.5 |  |  | | 51.5 | 42.7 |
| MedDiet+Nuts | 38.4 | 48.5 |  |  | | 48.5 | 57.3 |
| **Total workload of center (person-years)**^f^ | |  |  |  | |  |  |
| Q1 (low) | 20.8 | 13.4 |  |  | | 45.2 | 37.9 |
| Q2 | 24.6 | 25.1 |  |  | | 31.8 | 33.1 |
| Q3 | 29.1 | 18.3 |  |  | | 7.5 | 7.0 |
| Q4 (high) | 25.4 | 43.3 |  |  | | 15.5 | 22.0 |
| ^a^ Those randomized after November 2006 did not have the opportunity to provide information on 4-year adherence. ^b^ A validated MedDiet adherence assessment tool was used. 1 point was added for each item in adherence with the traditional MedDiet. High adherence = adherence with ≥11 items on 14-point dietary adherence score. Low adherence = adherence with <11 items. ^c^ Total CVD risk score was calculated by summing the following CVD risk factors: type 2 diabetes, hypertension, high blood cholesterol, family history of premature CHD, depression, obesity. ^d^ Tertiles of physical activity (MET-min/d): T1: <108; T2: 108-268; T3: ≥268. ^e^ Quartiles of energy intake (kcal/d), by sex: Men: Q1: <2051; Q2: 2051-<2394; Q3:2934-<2801; Q4: ≥2801. Women: Q1: <1786; Q2: 1786-<2109; Q3: 2109-<2465; Q4: ≥2465.  ^f^ Measured in quartiles of person years at center. After 1 Year: Q1: 133-<352; Q2: 352-<537; Q3: 537-<650; Q4: ≥650. | | | | | | | |

**Additional file 1: Table 6. Odds of high adherence with the MedDiet intervention at one and four years of follow-up^a^, with alternate representation of “total workload”.**

|  | **OR (95% CI) for dietary adherence**  **(≥11 vs. <11 points)^b^** | | | | | | | | | |
| --- | --- | --- | --- | --- | --- | --- | --- | --- | --- | --- |
|  | **1 Year** | | | | | **4 Years** | | | | |
| **Demographic Characteristics** | n | Crude | *p* | Multivariate | *p* | n | Crude | *p* | Multivariate | *p* |
| **Sex** |  |  |  |  |  |  |  |  |  |  |
| Men | 1820 | 1.00 (ref) |  | 1.00 (ref) |  | 1103 | 1.00 (ref) |  | 1.00 (ref) |  |
| Women | 2378 | 0.83 (0.73, 0.94) | 0.003 | 0.79 (0.64, 0.97) | 0.02 | 1350 | 0.76 (0.64, 0.90) | 0.001 | 0.92 (0.69, 1.23) | 0.58 |
| **Age at baseline** (years) |  |  |  |  |  |  |  |  |  |  |
| <65 | 1627 | 1.00 (ref) |  | 1.00 (ref) |  | 868 | 1.00 (ref) |  | 1.00 (ref) |  |
| ≥65 | 2571 | 1.01 (0.89, 1.14) | 0.90 | 0.98 (0.83, 1.15) | 0.79 | 1485 | 0.87 (0.74, 1.04) | 0.12 | 9.91 (0.73, 1.12) | 0.37 |
| **Educational level** |  |  |  |  |  |  |  |  |  |  |
| University or higher | 339 | 1.00 (ref) |  | 1.00 (ref) |  | 173 | 1.00 (ref) |  | 1.00 (ref) |  |
| Secondary school | 643 | 0.92 (0.71, 1.20) | 0.56 | 0.97 (0.73, 1.29) | 0.86 | 356 | 0.61 (0.42, 0.90) | 0.01 | 0.68 (0.46, 1.02) | 0.06 |
| Primary School | 3109 | 0.82 (0.65, 1.03) | 0.09 | 0.88 (0.73, 1.29) | 0.34 | 1768 | 0.64 (0.46, 0.90) | 0.009 | 0.82 (0.57, 1.18) | 0.28 |
| Less than primary school | 107 | 0.60 (0.39, 0.92) | 0.02 | 0.81 (0.50, 1.29) | 0.37 | 57 | 0.29 (0.15, 0.54) | <0.001 | 0.51 (0.26, 0.99) | 0.05 |
| **Occupation** |  |  |  |  |  |  |  |  |  |  |
| Retired | 2234 | 1.00 (ref) |  | 1.00 (ref) |  | 1127 | 1.00 (ref) |  | 1.00 (ref) |  |
| Working | 509 | 0.81 (0.67, 0.98) | 0.03 | 0.75 (0.59, 0.94) | 0.01 | 258 | 1.04 (0.89, 1.37) | 0.75 | 0.99 (0.72, 1.37) | 0.96 |
| Housewife | 1324 | 0.95 (0.83, 1.09) | 0.44 | 1.06 (0.89, 1.27) | 0.51 | 802 | 0.89 (0.74, 1.06) | 0.19 | 1.02 (0.79, 1.30) | 0.90 |
| Unemployed/unable to work | 131 | 0.64 (0.45, 0.91) | 0.01 | 0.70 (0.48, 1.04) | 0.08 | 66 | 0.87 (0.53, 1.44) | 0.59 | 1.01 (0.58, 1.73) | 0.99 |
| **Marital Status** |  |  |  |  |  |  |  |  |  |  |
| Married | 3266 | 1.00 (ref) |  | 1.00 (ref) |  | 1824 | 1.00 (ref) |  | 1.00 (ref) |  |
| Single | 157 | 0.97 (0.70, 1.34) | 0.85 | 1.02 (0.72, 1.44) | 0.93 | 88 | 1.08 (0.70, 1.68) | 0.73 | 0.94 (0.58, 1.51) | 0.79 |
| Widowed | 668 | 0.87 (0.74, 1.03) | 0.07 | 0.99 (0.82, 1.20) | 0.93 | 399 | 0.77 (0.62, 0.96) | 0.02 | 0.87 (0.67, 1.11) | 0.26 |
| Divorced or separated | 107 | 0.75 (0.51, 1.10) | 0.15 | 0.99 (0.65, 1.5) | 0.95 | 42 | 0.91 (.49, 1.69) | 0.76 | 1.17 (0.61, 2.27) | 0.64 |
| **Number of people in household** | 4198 | 1.00 (0.96, 1.05) | 0.89 | 1.02 (0.97, 1.07) | 0.54 | 2353 | 1.01 (0.94, 1.07) | 0.87 | 0.99 (0.93, 1.07) | 0.86 |
| **Health-Related Characteristics at Baseline** | | |  |  |  |  |  |  |  |  |
| **Number of CVD Risk Factors** ^c,d^ |  |  |  |  |  |  |  |  |  |  |
| 0-1 | 285 | 1.00 (ref) |  | 1.00 (ref) |  | 204 | 1.00 (ref) |  | 1.00 (ref) |  |
| 2 | 1237 | 0.89 (0.68, 1.15) |  | 0.95 (0.71, 1.25) |  | 734 | 0.76 (0.55, 1.05) |  | 0.82 (0.58, 1.17) |  |
| 3 | 1632 | 0.76 (0.59, 0.98) |  | 0.94 (0.71, 1.24) |  | 884 | 0.66 (0.48, 0.91) |  | 0.83 (0.58, 1.19) |  |
| 4 | 824 | 0.62 (0.47, 0.81) |  | 0.89 (0.65, 1.21) |  | 429 | 0.50 (0.35, 0.71) |  | 0.71 (0.48, 1.04) |  |
| 5-6 | 220 | 0.57 (0.40, 0.81) | <0.001 | 0.87 (0.59, 1.30) | 0.39 | 102 | 0.34 (0.21, 0.56) | <0.001 | 0.47 (0.27, 0.81) | 0.01 |
| **Type 2 Diabetes** |  |  |  |  |  |  |  |  |  |  |
| No | 2199 | 1.00 (ref) |  | 1.00 (ref) |  | 1221 | 1.00 (ref) |  | 1.00 (ref) |  |
| Yes | 1999 | 0.75 (0.66, 0.85) | <0.001 | 0.79 (0.68, 0.91) | 0.001 | 1132 | 0.71 (0.60, 0.84) | <0.001 | 0.74 (0.61, 0.90) | 0.002 |
| **Hypertension** |  |  |  |  |  |  |  |  |  |  |
| No | 757 | 1.00 (ref) |  | 1.00 (ref) |  | 447 | 1.00 (ref) |  | 1.00 (ref) |  |
| Yes | 3441 | 0.97 (0.83, 1.13) | 0.68 | 0.91 (0.76, 1.09) | 0.30 | 1906 | 0.87 (0.71, 1.08) | 0.21 | 0.78 (0.61, 1.00) | 0.05 |
| **Hypercolesterolaemia** |  |  |  |  |  |  |  |  |  |  |
| No | 1169 | 1.00 (ref) |  | 1.00 (ref) |  | 748 | 1.00 (ref) |  | 1.00 (ref) |  |
| Yes | 3029 | 1.14 (0.99, 1.30) | 0.06 | 0.91 (0.92, 1.24) | 0.41 | 1605 | 1.15 (0.96, 1.37) | 0.13 | 1.11 (0.91, 1.35) | 0.32 |
| **Family history of premature CHD** | |  |  |  |  |  |  |  |  |  |
| No | 3237 | 1.00 (ref) |  | 1.00 (ref) |  | 1871 | 1.00 (ref) |  | 1.00 (ref) |  |
| Yes | 961 | 0.89 (0.77, 1.02) | 0.10 | 0.89 (0.76, 1.04) | 0.15 | 482 | 0.64 (0.53, 0.79) | <0.001 | 0.63 (0.51, 0.79) | <0.001 |
| **Depression** |  |  |  |  |  |  |  |  |  |  |
| No | 3481 | 1.00 (ref) |  | 1.00 (ref) |  | 1975 | 1.00 (ref) |  | 1.00 (ref) |  |
| Yes | 717 | 0.87 (0.74, 1.02) | 0.10 | 0.99 (0.83, 1.18) | 0.91 | 378 | 0.79 (0.64, 0.99) | 0.04 | 0.80 (0.63, 1.02) | 0.08 |
| **Obesity** |  |  |  |  |  |  |  |  |  |  |
| No | 2282 | 1.00 (ref) |  | 1.00 (ref) |  | 1302 | 1.00 (ref) |  | 1.00 (ref) |  |
| Yes | 1916 | 0.78 (0.69, 0.88) | <0.001 | 0.90 (0.86, 0.94) | <0.001 | 1051 | 0.83 (0.70, 0.97) | 0.02 | 1.15 (0.92, 1.44) | 0.22 |
| **SBP** (per 5 mmHg) | 4198 | 1.01 (1.00, 1.03) | 0.07 | 1.02 (0.99, 1.02) | 0.56 | 2353 | 1.02 (1.00, 1.04) | 0.02 | 1.01 (099, 1.04) | 0.03 |
| **DBP** (per 5 mmHg) | 4198 | 1.02 (1.00, 1.05) | 0.09 | 1.03 (0.99, 1.06) | 0.17 | 2353 | 1.03 (0.99, 1.07) | 0.10 | 1.00 (0.95, 1.05) | 0.93 |
| **Waist circumference** (per 5 cm) | 4198 | 0.90 (0.88, 0.93) | <0.001 | 0.92 (0.88, 0.95) | <0.001 | 2353 | 0.90 (0.87, 0.94) | <0.001 | 0.96 (0.92, 1.01) | 0.12 |
| **Physical activity** (MET-min/d) ^c,e^ |  |  |  |  |  |  |  |  |  |  |
| T1 (low) | 1332 | 1.00 (ref) |  | 1.00 (ref) |  | 703 | 1.00 (ref) |  | 1.00 (ref) |  |
| T2 | 1414 | 1.28 (1.10, 1.48) |  | 1.18 (1.01, 1.39) |  | 785 | 1.46 (1.19, 1.80) |  | 1.30 (1.05, 1.62) |  |
| T3 (high) | 1452 | 1.70 (1.46, 1.98) | <0.001 | 1.47 (1.25, 1.74) | <0.001 | 865 | 2.12 (1.72, 2.60) | <0.001 | 1.67 (1.33, 2.10) | <0.001 |
| **Smoking Status** |  |  |  |  |  |  |  |  |  |  |
| Never | 2576 | 1.00 (ref) |  | 1.00 (ref) |  | 1482 | 1.00 (ref) |  | 1.00 (ref) |  |
| Former | 574 | 0.90 (0.75, 1.08) | 0.24 | 0.82 (0.66, 1.03) | 0.09 | 320 | 1.16 (0.90, 1.48) | 0.25 | 0.96 (0.71, 1.30) | 0.80 |
| Current | 1048 | 1.17 (1.01, 1.35) | 0.04 | 1.07 (0.89, 1.29) | 0.48 | 551 | 1.17 (0.95, 1.42) | 0.13 | 1.01 (0.78, 1.30) | 0.96 |
| **Total energy intake** (kcal/day) ^c, f^ |  |  |  |  |  |  |  |  |  |  |
| Q1 (low) | 1017 | 1.00 (ref) |  | 1.00 (ref) |  | 517 | 1.00 (ref) |  | 1.00 (ref) |  |
| Q2 | 1038 | 1.31 (1.10, 1.56) |  | 1.27 (1.06, 1.53) |  | 551 | 1.17 (0.92, 1.49) |  | 1.08 (0.84, 1.40) |  |
| Q3 | 1078 | 1.34 (1.13, 1.59) |  | 1.23 (1.02, 1.47) |  | 596 | 1.41 (1.11, 1.79) |  | 1.27 (0.98, 1.64) |  |
| Q4 (high) | 1065 | 1.50 (1.26, 1.79) | <0.001 | 1.44 (1.20, 1.74) | <0.001 | 689 | 1.36 (1.08, 1.72) | 0.007 | 1.26 (0.98, 1.62) | 0.001 |
| **Alcohol other than wine** (g/day) |  |  |  |  |  |  |  |  |  |  |
| <10 men, <5 women | 2429 | 1.00 (ref) |  | 1.00 (ref) |  | 1360 | 1.00 (ref) |  | 1.00 (ref) |  |
| 10-50 men, 5-10 women | 606 | 1.04 (0.87, 1.24) | 0.67 | 0.85 (0.69, 1.04) | 0.11 | 344 | 1.03 (0.81, 1.31) | 0.81 | 0.82 (0.63, 1.08) | 0.16 |
| >50 men, >10 women | 1163 | 1.00 (0.87, 1.15) | 0.97 | 0.92 (0.78, 1.07) | 0.27 | 649 | 1.09 (0.90, 1.32) | 0.38 | 1.03 (0.84, 1.28) | 0.76 |
| **14-point adherence score** ^a^ | | |  |  |  |  |  |  |  |  |
| <11 | 3416 | 1.00 (ref) |  | 1.00 (ref) |  | 1864 | 1.00 (ref) |  | 1.00 (ref) |  |
| ≥11 | 782 | 3.41 (2.85, 4.07) | <0.001 | 3.07 (2.56, 3.70) | <0.001 | 489 | 2.06 (1.66, 2.6) | <0.001 | 1.80 (1.43, 2.26) | <0.001 |
| **Study Design Features** |  |  |  |  |  |  |  |  |  |  |
| **Intervention Group** |  |  |  |  |  |  |  |  |  |  |
| Nuts | 1962 | 1.00 (ref) |  | 1.00 (ref) |  | 1027 | 1.00 (ref) |  | 1.00 (ref) |  |
| Olive Oil | 2236 | 0.70 (0.62, 0.79) | <0.001 | 0.70 (0.62, 0.80) | <0.001 | 1326 | 0.74 (0.62, 0.87) | <0.001 | 0.75 (0.63, 0.89) | 0.05 |
| **Total workload of center (number of participants)**^e,g^ | | |  |  |  |  |  |  |  |  |
| <300 | 395 | 1.00 (ref) |  | 1.00 (ref) |  | 431 | 1.00 (ref) |  | 1.00 (ref) |  |
| 300 to <450 | 1661 | 1.42 (1.13, 1.79) |  | 1.37 (1.07, 1.75) |  | 554 | 1.11 (0.86, 1.43) |  | 1.16 (0.89, 1.52) |  |
| 450 to <600 | 897 | 1.08 (0.84, 1.38) |  | 1.00 (0.77, 1.30) |  | 804 | 0.95 (0.75, 1.20) |  | 1.03 (0.81, 1.32) |  |
| ≥600 | 1281 | 2.71 (2.13, 3.44) | <0.001 | 2.52 (1.95, 3.26) | <0.001 | 564 | 3.00 (2.28, 3.94) | <0.001 | 2.71 (2.03, 3.61) | <0.001 |
| ^a^ Control group was excluded. ORs < 1 imply poorer adherence. ORs > 1 imply better adherence. A validated MedDiet adherence assessment tool was used. 1 point was added for each item in adherence with the traditional MedDiet. High adherence = adherence with ≥11 items on 14-point dietary adherence score. Low adherence = adherence with <11 items. ^b^ All models are from logistic regression analysis. Multivariate models are mutually adjusted for all characteristics displayed in this table including total CVD risk score but excluding individual CVD risk factors (type 2 diabetes, hypertension, hypercholesterolaemia, family history of pre-mature CHD, depression, obesity). When an individual CVD risk factors was the exposure of interest, the model was mutually adjusted for other individual CVD risk factors but not total CVD risk score. ^c^ P-values for trend were calculated by assigning the median value to each category and treating the resulting variable as continuous. ^d^ Risk score calculated by summing the following CVD risk factors: type 2 diabetes, hypertension, high blood cholesterol, family history of premature CHD, depression, obesity. ^e^ Tertiles of physical activity (MET-min/d): T1: <108; T2: 108-268; T3: ≥268. ^f^ Quartiles of energy intake (kcal/d), by sex: Men: Q1: <2051; Q2: 2051-<2394; Q3:2934-<2801; Q4: ≥2801. Women: Q1: <1786; Q2: 1786-<2109; Q3: 2109-<2465; Q4: ≥2465. ^g^ Measured in number of participants per center (<300, 300-<450, 450-<600, ≥600). | | | | | | | | | | |
