# Supplementary figures and images for: Predictors of short- and long-term adherence with a Mediterranean-type diet intervention: the PREDIMED randomized trial
Source: Int J Behav Nutr Phys Act. 2016 Jun 14;13:67. doi: 10.1186/s12966-016-0394-6 (PMC4907003; doi:10.1186/s12966-016-0394-6)

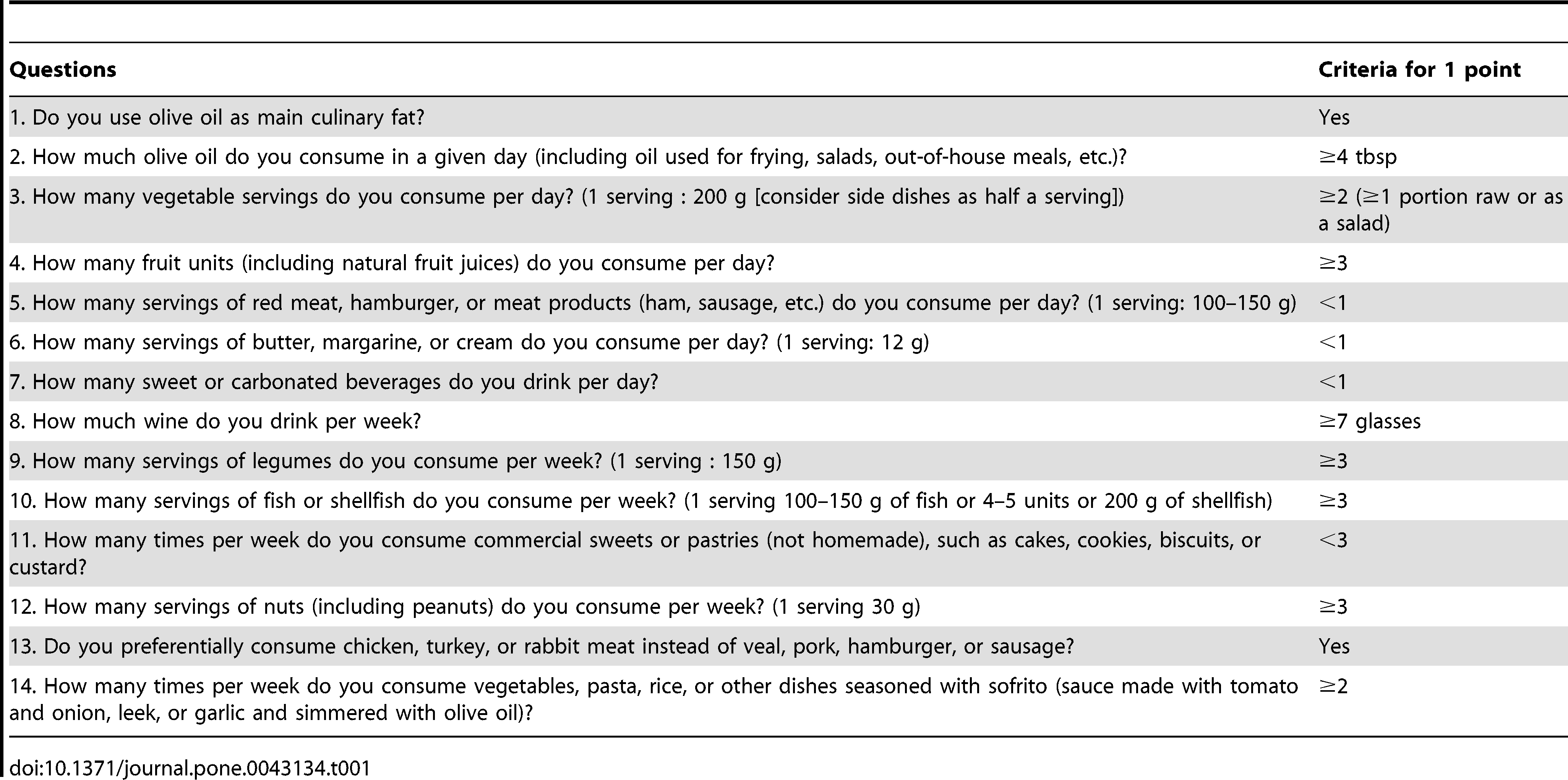

Supplement: Additional file 2: — Figure S1. Validated 14-item questionnaire of mediterranean diet adherence (DOCX 205 kb) [file 12966_2016_394_MOESM2_ESM.docx]
